# NPR1 Translocation from Chloroplast to Nucleus Activates Plant Tolerance to Salt Stress

**DOI:** 10.3390/antiox12051118

**Published:** 2023-05-18

**Authors:** Soyeon Seo, Yumi Kim, Kyyoung Park

**Affiliations:** Department of Biomedical Science, Sunchon National University, Suncheon 57922, Jeollanam-do, Republic of Korea; soyeon@scnu.ac.kr (S.S.); 1220025@s.scnu.ac.kr (Y.K.)

**Keywords:** stress tolerance, retrograde signaling, NPR1, transcription coactivator, chloroplast, redox, *Nicotiana tabacum*

## Abstract

Chloroplasts play crucial roles in biotic and abiotic stress responses, regulated by nuclear gene expression through changes in the cellular redox state. Despite lacking the N-terminal chloroplast transit peptide (cTP), nonexpressor of pathogenesis-related genes 1 (NPR1), a redox-sensitive transcriptional coactivator was consistently found in the tobacco chloroplasts. Under salt stress and after exogenous application of H_2_O_2_ or aminocyclopropane-1-carboxylic acid, an ethylene precursor, transgenic tobacco plants expressing green fluorescent protein (GFP)-tagged NPR1 (NPR1-GFP) showed significant accumulation of monomeric nuclear NPR1, irrespective of the presence of cTP. Immunoblotting and fluorescence image analyses indicated that NPR1-GFP, with and without cTP, had similar molecular weights, suggesting that the chloroplast-targeted NPR1-GFP is likely translocated from the chloroplasts to the nucleus after processing in the stroma. Translation in the chloroplast is essential for nuclear NPR1 accumulation and stress-related expression of nuclear genes. An overexpression of chloroplast-targeted NPR1 enhanced stress tolerance and photosynthetic capacity. In addition, compared to the wild-type lines, several genes encoding retrograde signaling-related proteins were severely impaired in the *Arabidopsis npr1-1* mutant, but were enhanced in NPR1 overexpression (*NPR1-Ox*) transgenic tobacco line. Taken together, chloroplast NPR1 acts as a retrograding signal that enhances the adaptability of plants to adverse environments.

## 1. Introduction

A fundamental question in plant physiology and cell biology is the communication between organelles and the nucleus, which coordinates genetic programs and cellular functions [1]. In all organisms, including plants, redox-associated signaling is an essential component of responses to environmental stresses and pathogen attacks [2]. In plants, stress-related reactive oxygen species (ROS) and redox signals mainly accumulate in the chloroplasts.

The communication between chloroplasts and the nucleus occurs through retrograde signaling, which involves an ROS-mediated redox cascade. This cascade activates protective mechanisms and modulates hormonal biosynthesis [3,4]. Proteins that are sensitive to ROS and catalyze the oxidation of cysteine residues act as redox switches in response to abiotic and biotic stresses [5]. Furthermore, the disruption of photosynthetic activity and metabolite production in chloroplasts under environmental stress or pathogen attack induces multiple homeostatic pathways mediating protein folding and degradation [6]. Thus, proteostasis must be maintained in the chloroplasts by preventing protein misfolding and aggregation to ensure proper cell function [7,8]. Although the chloroplast-to-nucleus retrograde signaling pathway under environmental stress-adaptive response and during chloroplast development has been considerably explored, the translocation mechanisms of metabolite or protein remain poorly understood. One potential mechanism is that proteins with cysteine residues can act as retrograde signaling switches through redox-sensitive post-translational modifications [9,10,11].

The nonexpressor of pathogenesis-related genes 1 (NPR1) acts as a transcriptional coactivator and is a key component of plant immunity through salicylic acid (SA)-mediated defense responses and systemic-acquired resistance (SAR) in *Arabidopsis thaliana* [12]. Pathogen-induced SA triggers changes in cellular reduction potential that convert the cytoplasmic NPR1 tetramer into a monomer, which is then transported into the nucleus to function as a transcription coactivator [13]. The degradation of nuclear NPR1 results in a transient immune response that ultimately confers fitness. Despite previous studies having focused on the transcriptional coactivator role of NPR1 in *Arabidopsis*, our previous work has shown that NPR1, which accumulates in chloroplasts under salt stress, exhibits antioxidant and chaperone activities [7]. In this study, we investigated the functions of chloroplast NPR1 and its translocation to the nucleus based on its recently reported structural characteristics. Our investigation focused on determining whether chloroplast NPR1 is a mobile molecule that can transmit signals of abiotic stress, recognized by chloroplasts, to the nucleus, as well as identifying the factors that affect its migration.

## 2. Materials and Methods

### 2.1. Plant Material and Growth Conditions

*Nicotiana tabacum* Wisconsin-38 was used as the wild-type (WT) plant to create the transgenic lines. *Arabidopsis thaliana* Col-0 WT and *npr1-1* mutant (Arabidopsis Biological Resource Center, Ohio State University, Columbus, OH, USA) were used in this study. Tobacco and *Arabidopsis* seeds were cultured on half-strength Murashige and Skoog medium under photoperiod (16L/8D, 100 μmol photons m^−2^ s^−1^) at 25 °C. The seedlings were transferred to soil as required, and grown for 3–5 weeks under 16L/8D light cycles. Fully mature WT and transgenic plants were subjected to various chemical treatments, including salt stress. The tobacco plants were treated with 200 mM NaCl, while the *Arabidopsis* plants were treated with 100 mM NaCl.

### 2.2. Gene Constructs and Transgenic Plants

*p35S::NPR1-GFP* and *p35S::NPR1-Ox* transgenic plants were previously described [7]. The open reading frame of *NPR1* was amplified by polymerase chain reaction (PCR) and cloned into a *pMBP* vector containing the *35S* promoter-driven green fluorescent protein (GFP) gene and *NOS terminator*. The native *NPR1* promoter was amplified via PCR from genomic DNA of *N. tabacum*. To generate *pNPR1::NPR1-GFP* transgenic plants, a 0.8 kbp fragment of the *NPR1* promoter was amplified via PCR and cloned into a promoter-less *NPR1-GFP* construct, which was obtained by deleting the *35S* promoter region from *p35S::NPR1-GFP*. To generate *p35S::cTP-NPR1-GFP* transgenic plants, a 237 bp PCR product of the chloroplast transit peptide (cTP; 79 amino acid residues) from the small subunit of RuBisCo (GenBank AY220079) was cloned between the end of the *35S* promoter and the 5′-end of *NPR1* in the *p35S::NPR1-GFP* construct. To generate the *p35S::NPR1(∆nls)-GFP* construct, a 54 bp nuclear localization sequence (NLS) from nucleotide position 1612 to 1665 in the *NPR1* gene was deleted from *p35S::NPR1-GFP* using the GeneArt Site-directed Mutagenesis PLUS kit (Thermo Fisher Scientific, Waltham, MA, USA). The *NPR1-GFP* constructs were cloned into *pBI121* and utilized for the generation of transgenic plants. The transgenic plants were advanced to the T3 generation. Despite being T3 homozygous, they were used as an experimental plant after confirming kanamycin resistance. 

### 2.3. RNA Isolation and Real-Time RT-PCR

Total RNA was isolated using TRIzol reagent (Molecular Research Center, Cincinnati, OH, USA). For the analysis of relative transcript levels using real-time RT-PCR,3 μg, the total RNA from leaves was reverse-transcribed using the high-fidelity PrimeScript^TM^ cDNA Synthesis Kit (Takara, Japan) in a 20 μL reaction volume at 42 °C for 30 min, following the instructions of the manufacturer. Gene-specific PCR primers for qPCR were designed based on sequence information from the GenBank database, using stringent criteria (Table S1): a predicted melting temperature of 60 ± 5 °C, a primer length of 20–24 nucleotides, 50–60% guanine-cytosine content, and a PCR fragment length of 100–250 bp. The PCR efficiency of primers was confirmed to be over 95% using an efficiency calculator, based on a 6-point dilution with a 10-fold series of cDNA. Real-time PCR was performed in optical 96-well plates with iQ^TM^ SYBR Green Super Mix (Bio-RAD, USA), using a TP950 thermal cycler (Takara, Japan). Real Time System III Software version 6.0 (Takara, Japan) was used, and data was exported to Microsoft Excel. The transcription levels were expressed relative to the reference gene (β-actin) level. Expression analysis was performed using three independent triplicate experiments to determine the mean relative transcript expression levels for each gene in the WT, *NPR1* overexpression (*NPR1-Ox*) transgenic plants, and *npr1-1* mutants. The expression ratio of each gene was calculated by comparing the WT versus *NPR1-Ox* or Col-0 WT versus *npr1-1* mutants. The transcription profiles of the genes involved in retrograde communication for chloroplast development and operational signaling in response to salt stress were determined.

### 2.4. Trypan Blue Staining

To assess plant cell death, tobacco plants treated with salt stress were immersed in a boiling solution containing 10 mL lactic acid, 10 mL glycerol, 10 g phenol, and 0.4% (*w*/*v*) trypan blue for 1 min. After cooling to room temperature, the plants were submerged in 70% (*w*/*v*) chloral hydrate. The stained plants were decolorized overnight and then photographed using a digital camera.

### 2.5. Analysis of Photosynthetic Activity

To measure steady-state net photosynthesis, eight-week-old plants were subjected to a gas exchange measuring station (Walz, Germany) equipped with a built-in light source (210 μmol photons m^−2^ s^−1^). A gas stream (60 L h^−1^, 21% O_2_ and 430 μL^−1^ CO_2_) was continuously supplied to the photosynthesis unit using a mass flow control system. The temperature inside the growth chamber was maintained at 25 °C, and the humidity was kept at 70 ± 1%.

### 2.6. GFP and CFP Detection

The expression of NPR1 tagged with either GFP (NPR1-GFP) or cyan fluorescent protein (CFP) (NPR1-CFP) was investigated in both intact leaves and protoplasts of stable *NPR1-GFP* transgenic plants. Confocal laser scanning microscopes (A1 HD25 from Nikon, Japan; FluoView 300 from Olympus, Tokyo, Japan; and STELLARIS 8 from Leica, Wetzlar, Germany) and a fluorescence microscope (THUNDER Imager from Leica, Wetzlar, Germany) equipped with a high-resolution CCD camera (A1 HD25 from Nikon, Tokyo, Japan) were used to visualize the GFP and CFP fluorescence in the cells. Excitation and emission were set at 488 and 520 nm, respectively, for GFP, and at 450 and 470 nm, respectively, for CFP. Red chlorophyll fluorescence was visualized by exciting at 458 nm and a detecting emission at 647–720 nm. The intensity of fluorescence was quantified using the Image J software (https://imagej.nih.gov/ij/, accessed on 15 March 2023). Thirty images were acquired per independent experiment, and each experiment was repeated more than three times *(n* = 30, *N* > 3*).*

### 2.7. ROS Detection in Leaves

To determine the total amount of ROS, epidermal strips were peeled from tobacco leaves after exposure to salt stress for different time intervals, and immersed in a 50 μM 2′,7′-dichlorofluorescin diacetate (DCFH-DA) solution (Sigma-Aldrich; St Louis, MO, USA). ROS levels were observed using a fluorescence microscope (excitation = 450–490 nm; barrier = 520–560 nm) equipped with a CCD camera (FluoView 300, Olympus, Tokyo, Japan). The O_2_^−^ and H_2_O_2_ levels were determined using 0.2% nitroblue tetrazolium solution in 50 mM sodium phosphate buffer (pH 7.5) and 1 mg mL^−1^ diaminobenzidine staining solution in distilled water, respectively.

### 2.8. Chloroplast and Nucleus Isolation, Protein Extraction, and Western Blotting

For chloroplast isolation, intact leaves were used and a chloroplast isolation kit (Sigma-Aldrich, Saint Louis, MO, USA) was employed. Intact chloroplasts were collected using a 40/80% Percoll gradient. The chloroplasts were suspended in a chloroplast lysis buffer containing 0.5 mM HEPES–KOH (pH 7.5), 2 mM MgCl_2_, 1 mM NaF, 1 mM EDTA, 1 mM phenylmethylsulfonyl fluoride, 80 μM MG115, 80 μM MG132, and a protease inhibitor cocktail tablet (Roche Molecular Biochemicals, Basel, Switzerland). After centrifugation of the lysate at 15,000× *g* for 15 min, the supernatant was collected as a chloroplast stroma protein fraction.

Nuclear proteins were extracted using a plant nuclei isolation/extraction kit (CelLyticTM PN; Sigma-Aldrich, Saint Louis, MO, USA), following the manufacturer’s instructions. First, intact leaves were incubated with the nuclei isolation buffer, and then the resulting cell lysate was filtered using a mesh. A pure preparation of the nuclei was obtained via centrifugation at 12,000× *g* for 10 min, using 2.3 M sucrose. Nuclear proteins were extracted from the pellet using the Working Extraction Buffer, and then centrifuged to obtain a pure supernatant as the soluble nuclear protein. 

To extract cytosolic proteins from tobacco plants, frozen leaves were homogenized with a homogenization buffer containing 50 mM HEPES–KOH (pH 7.5), 250 mM sorbitol, 50 mM potassium acetate, 2 mM magnesium acetate, 1 mM EDTA, 1 mM EGTA, 1 mM DTT, 80 μM MG115, 80 μM MG132, and a protease inhibitor cocktail tablet. The crude nuclear and chloroplast fractions were removed via centrifugation at 1000× *g* for 15 min, and the mitochondrial fraction was removed at 20,000× *g* for 15 min. The final supernatant was obtained as the crude cytosolic fraction. 

The proteins were separated using 4–12% or 3–8% Bis-Tris Plus gels, and transferred onto iBlot 2 NC Regular Stacks (Invitrogen, Carlsbad, CA, United States). Ponceau S staining was performed to ensure equal loading. The blots were blocked using iBind Cards (Novex), according to the manufacturer’s instructions. NPR1-GFP proteins were detected by incubating the blots with a mouse monoclonal anti-GFP antibody (Clontech, Mountain View, CA, USA). Anti-Toc75 (Agrisera, Vännäs, Sweden) antibodies were used as the marker for the chloroplast outer membrane, while anti-RbcL (Agrisera) or anti-histon3 (Agrisera) primary antibodies were used as markers for the chloroplast stroma and the nucleus, respectively. The bands were visualized using West Glow^TM^ FEMTO Chemiluminescent Sustrate (BioMax, Guri, Republic of Korea) on X-ray film. All experiments were repeated at least three times, and the representative data are presented. The intensity of the protein bands was quantified using the Image J software (v1.54d, Bethesda, MD, USA).

### 2.9. Transient Expression in Tobacco Protoplasts

Mesophyll protoplasts were isolated from both WT and transgenic tobacco leaves using enzyme solution containing 0.5 M mannitol, 1 mM CaCl_2_, 20 mM MES, 0.1% BSA, 1% cellulase R-10, and 0.25% macerozyme R-10. After 30 min stabilization on ice, 10^6^ mL^−1^ protoplasts were used for further transient transformation. Protoplasts (300 µL) in W5 buffer were gently pipetted into a pre-chilled 0.4 cm electroporation cuvette, and 50 µg of the plasmid construct in 10 µL TE buffer was added. Electroporation was performed using the Gene Pulser Xcell System (Bio-Rad, Hercules, CA, USA) at 160 V/960 μF (voltage/capacitance), according to the manufacturer’s instructions. The protoplasts were then transferred to a conical tube containing 500 μL K3 medium (154 mM NaCl, 125 mM CaCl_2_, 5 mM sucrose, 5 mM xylose, and 1.5 mM MES (pH 5.7)) using a glass Pasteur pipette, and observed using confocal microscopes (STELLARIS 8 and A1 HD25, Leica, Wetzlar, Germany).

### 2.10. Statistical Analyses

The experiments were repeated at least three times with three replicates each time. The data from a representative experiment are presented after confirming that similar results were obtained. Statistical analyses were performed using a paired two-sample Student’s t-test, and the data are presented as the mean ± standard deviation (SD). The figure legends provide detailed statistical parameters, including *p*-values and statistical significance. At each time point, statistically significant differences between wild-type and transgenic plant lines or each treatment group, and the corresponding control group are indicated by * *p* < 0.05 or ** *p* < 0.01. 

## 3. Results

### 3.1. NPR1 Accumulation in the Chloroplast and Nucleus under Salt Stress

**Figure 1:**
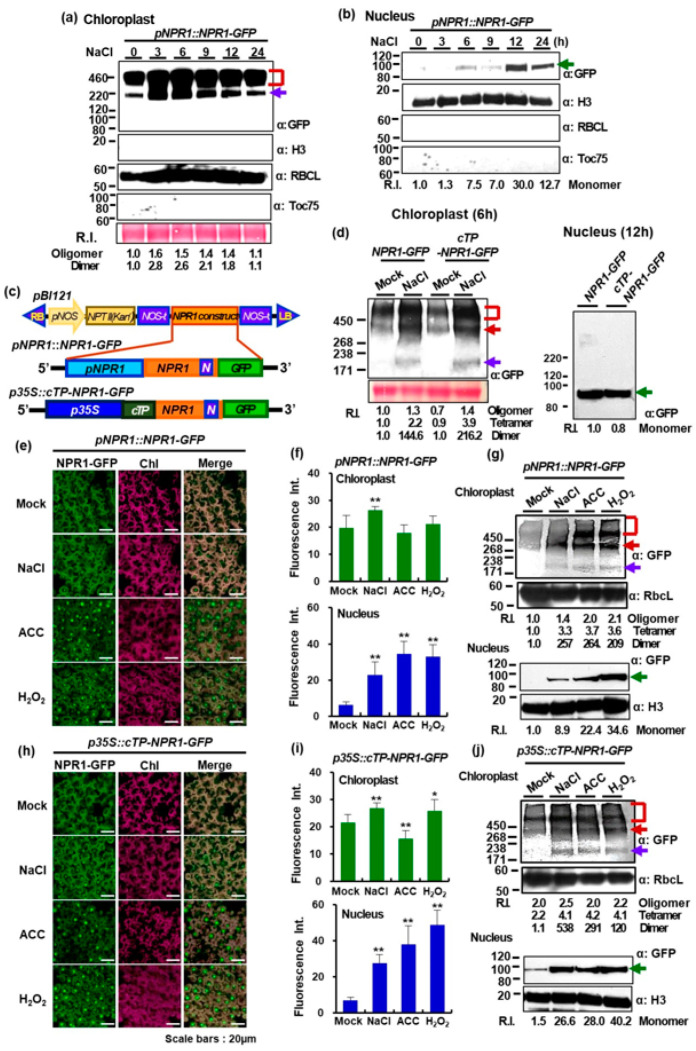
Intracellular localization of green fluorescent protein-tagged nonexpressor of pathogenesis-related genes 1 (GFP-NPR1) in leaf cells under salt stress. (**a**,**b**) Immunoblots showing GFP-tagged NPR1 (NPR1-GFP) in the protein fractions of the chloroplasts (**a**) and nucleus (**b**) from *pNPR1::NPR1-GFP* transgenic plants under salt stress using non-denatured SDS–PAGE (4–12% gradient polyacrylamide *gel*). Oligomers (red square bracket), the dimeric form (purple arrow), and monomeric form (green arrow). Ponceau S was used to assess equal loading among the samples. Relative intensity was calculated by normalizing the band intensity values to the 0 h treatment, which was assigned as 1.0. Anti-Histone H3 antibody (H3): marker of nuclear proteins; Anti-RbcL antibody (RBCL): marker of chloroplast stroma proteins; anti-Toc75 antibody (Toc75): marker of chloroplast outer membrane. (**c**) Schematic diagram of the *NPR1* construct introduced into *pBI121* for transgenic plants. (**d**) Immunoblot analysis of proteins isolated from chloroplasts (6 h) and nuclei (12 h) of 6-week-old soil-grown *pNPR1::NPR1-GFP* or *p35S::cTP-NPR1-GFP* transgenic plants after salt stress, using a non-denatured gradient SDS–PAGE (chloroplast: 3–8%; nucleus: 4–12%*)*. (**e**,**h**) Confocal laser scanning microscopy (CLSM) images of GFP fluorescence in mesophyll cells from both (**e**) *pNPR1::NPR1-GFP* or (**h**) *p35S::cTP-NPR1-GFP* transgenic plants treated with 200 mM NaCl, 1 mM 1-amino-1-cyclopropane carboxylic acid (ACC), or 5 mM H_2_O_2_. Images of (green) GFP fluorescence and (magenta) chlorophyll autofluorescence from chloroplasts are merged in the third column. (**f**,**i**) Fluorescence intensity of NPR1-GFP in the (upper panel) chloroplasts and (lower panel) nucleus of (**f**) *pNPR1::NPR1-GFP* or (i) *p35S::cTP-NPR1-GFP* transgenic plants treated with 200 mM NaCl, 1 mM ACC, or 5 mM H_2_O_2_. GFP fluorescence was visualized in the mesophyll cells of transgenic plants following salt, ACC, or H_2_O_2_ application, and the GFP intensity was quantified in the chloroplasts and the nucleus using Image J (*n* = 30). Data are expressed as the mean ± SD. Asterisk indicates significant differences between the mock treatment with salt and other chemicals (* *p* < 0.05 or ** *p* < 0.01). (**g**,**j**) Immunoblots showing NPR1–GFP in the (upper panel) chloroplast and (lower panel) nucleus protein fractions from (**g**) *pNPR1::NPR1-GFP* and (**j**) *p35S::cTP-NPR1-GFP* transgenic plants treated with NaCl, ACC, or H_2_O_2_ using non-denatured SDS–PAGE. In Western blot analysis, 3–8% and 4–12% were used as gradient gels for the chloroplast and nuclear proteins, respectively. Oligomers (red square bracket and arrow), the dimeric form (purple arrow), and monomeric form (green arrow). The relative band intensities were determined using the Image J software, with the control value set to 1.

Previously, we generated stable transgenic tobacco plants expressing a fusion construct of full-length *NPR1* and *GFP* driven by the *NPR1* promoter (*pNPR1::NPR1-GFP*) [7]. Although the NPR1 is a well-known transcription factor [12], it was present at significant amounts in chloroplasts, regardless of stress. Western blot analysis detected approximately 200, 400, and 600 kDa NPR1-GFP oligomers (dimers, tetramers, and higher) in the chloroplast protein fractions from *pNPR1::NPR1-GFP* transgenic tobacco plants after salt treatment (Figure 1a). The amounts of dimeric and tetrameric forms increased after salt stress, but gradually decreased after 9 h. However, after 12–24 h of salt stress, only monomeric NPR1 was detected in the nuclear protein fraction of mesophyll cells (Figure 1b).

We attached the 9.8 kDa *cTP* sequence (79 amino acid residues) of tobacco *RbcS* (GenBank accession number AY220079) to the 5′ end of NPR1-GFP to generate *p35S::cTP-NPR1-GFP* transgenic tobacco lines (Figure 1c). We used immunoblotting analysis to investigate the presence of the NPR1-GFP fusion protein in the chloroplasts and nuclei of soil-grown leaves from both transgenic lines 6 and 12 h after salt stress, respectively. Specifically, a lower-percentage gel was used for the chloroplast fraction to resolve larger NPR1 molecules. The dual localization patterns of the GFP fusion proteins were similar under salt stress in both lines (Figure 1d). Notably, the molecular weight of monomeric nuclear NPR1-GFP in the *cTP-NPR1-GFP* line was identical to that in the *NPR1-GFP* line (approximately 92.3-kDa) (Figure 1d). The introduced the chloroplast-targeted NPR1 was predicted to be the size of cTP-NPR1-GFP (102.1 kDa), but the size of the NPR1 protein detected in the nucleus was the same as the cTP-cleaved NPR1-GFP, which was 92.3-kDa. This suggests that nuclear NPR1-GFP does not contain cTP, and therefore does not directly enter the nucleus from the cytosol after translation. The cTP attached to the N-terminus of NPR1-GFP is cleaved off in the chloroplast and then migrated to the nucleus. This finding indicates that salt stress induced NPR1 translocation from the chloroplasts to the nucleus after cTP cleavage in the stroma by the stromal processing peptidase [14,15]. 

We further investigated the cellular localization of NPR1 after salt stress or treatment with H_2_O_2_ and 1-amino-1-cyclopropane carboxylic acid (ACC), an ethylene precursor, using confocal laser scanning microscopy (CLSM; STELLARIS 8 and A1 HD25). Fluorescence of GFP alone was consistently detected in the cytoplasm and nucleus of *35S::GFP* transgenic plants, and it did not merge with red autofluorescence from chloroplasts, indicating that GFP rarely entered the chloroplasts in tobacco leaves (Figure S1). In contrast, NPR1-GFP fluorescence was found to be enhanced in the chloroplasts of mesophyll cells of *pNPR1::NPR1-GFP* transgenic tobacco plants after 200 mM NaCl-induced salt stress (Figure 1e), as confirmed by quantification using Image J (Figure 1f). This indicates that NPR1 has a specific region involved in NPR1-GFP protein migration into chloroplasts.

We found that NPR1 was present at significant amounts in chloroplasts, even without stress. Western blot analysis showed the presence of approximately 180, 370, and larger-than-500 kDa NPR1-GFP oligomers (dimers, tetramers, and higher) in the chloroplast protein fractions from *pNPR1::NPR1-GFP* transgenic tobacco leaves 6 h after treatments (Figure 1g and Figure S2). The amounts of dimeric and tetrameric forms increased in chloroplast fraction, but only monomeric NPR1 was detected in the nuclear protein fractions of mesophyll cell under salt stress (Figure 1g).

To investigate the dependence of subcellular NPR1 accumulation on stress-related signaling molecules, the spatial pattern of NPR1-GFP was analyzed in ACC- or H_2_O_2_-treated leaves. The fluorescent NPR1 signals in chloroplasts were significantly enhanced after both treatments (Figure 1e and Figure S3). After 6 h of treatment with either H_2_O_2_ or ACC, NPR1-GFP fluorescence was also clearly observed in the nuclei of mesophyll cells of *pNPR1::NPR1-GFP* transgenic plants. The fluorescence intensity of NPR1-GFP was greater in the nuclei of mesophyll cells of transgenic plants after H_2_O_2_ or ACC treatment compared to the untreated control and salt-stressed plants (Figure 1f). Moreover, the molecular weight of oligomeric, dimeric, tetrameric, and higher chloroplast NPR1-GFP proteins significantly increased in the transformant leaves following both applications (Figure 1g). Additionally, monomeric nuclear NPR1 significantly accumulated after 6 h of both treatments with ACC or H_2_O_2_.

### 3.2. Chloroplast-Targeted NPR1 Migration under Salt Stress

NPR1 accumulates in the nucleus, where it functions as a transcriptional coactivator during SAR [12,13]. We further investigated whether the NPR1 is translocated from the chloroplast to the nucleus, as the cTP-NPR1-GFP, which is targeted to the chloroplast by cTP, had the same molecular weight in both the chloroplast and nuclear protein fractions (Figure 1a,b,d). Furthermore, based on the temporal and spatial patterns, the levels of dimeric and tetrameric NPR1 slightly decreased in the chloroplast, while the levels of NPR1 monomers increased in the nucleus, suggesting that NPR1 is likely to be transported to the nucleus.

After exposing transgenic plants (*p35S::cTP-NPR1-GFP*) to salt stress or exogenous ACC or H_2_O_2_ application, the GFP fluorescence levels of nuclear NPR1 from chloroplast-targeted NPR1-GFP appeared distinctly in mesophyll cells (Figure 1h). The fluorescence intensity of nuclear NPR1-GFP increased significantly in the mesophyll cells of transformants after treatment with H_2_O_2_ or ACC, compared to that under salt stress (Figure 1i). Additionally, the levels of tetrameric and dimeric NPR1-GFP proteins significantly increased in the leaves of *p35S::cTP-NPR1-GFP* after both treatments (Figure 1j).

Notably, the abundance of monomeric NPR1 significantly increased in the nuclear fractions under salt stress and after exogenous ACC or H_2_O_2_ application (Figure 1g,j), indicating that the redox-sensitive NPR1 was reduced due to conformational changes in response to salinity stress and stress signaling. Moreover, in the chloroplasts of both transformants, stress-induced ROS and ethylene-promoted NPR1 dimerization, which is more favorable for movement.

**Figure 2:**
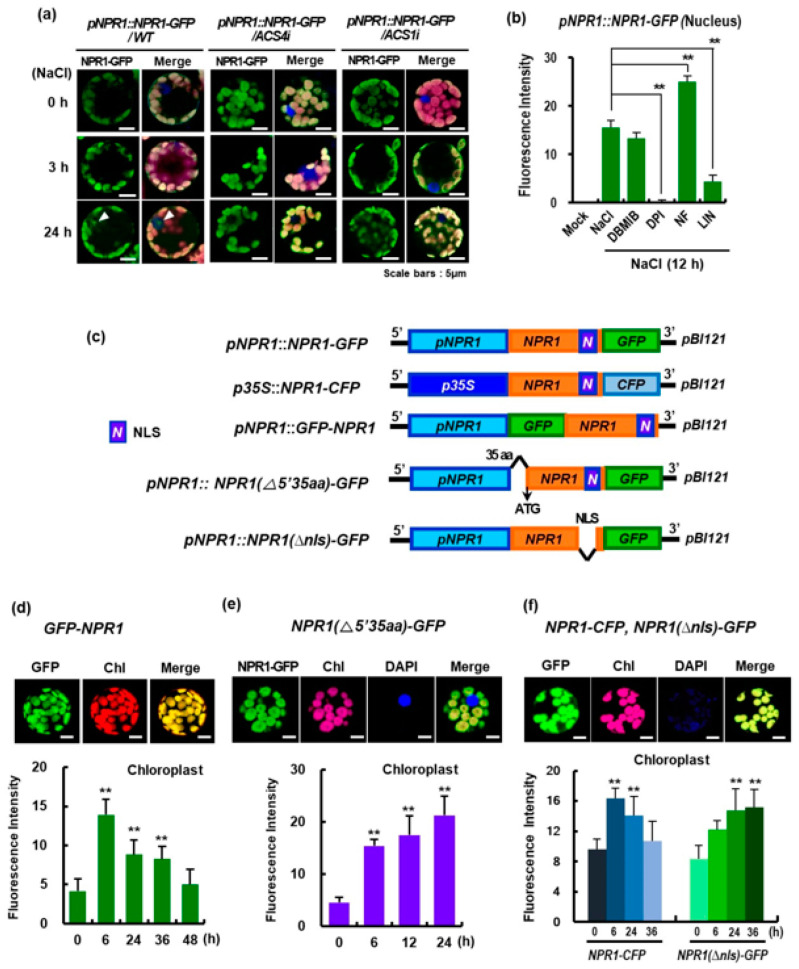
The green fluorescent protein (GFP) fluorescence levels in tobacco protoplasts after transient expression with mutated NPR1 constructs. (**a**) CLSM images of GFP fluorescence in mesophyll protoplasts from transient *pNPR1::NPR1-GFP*-expressing wild-type (WT) or ACC synthase (ACS)-RNA interference (RNAi) lines (*NtACS4i* and *NtACS1i*). Following transient expression, WT plants and RNAi lines were exposed to salt stress with 200 mM NaCl. Images of GFP fluorescence (green, first column) are merged with chlorophyll autofluorescence from the chloroplasts (magenta) in the second column. (**b**) NPR1−GFP fluorescence intensity in the nucleus of *p35S::NPR1-GFP* transgenic plants under salt stress. The mesophyll protoplasts were co-treated with inhibitors and NaCl. Inhibitors: DBMIB, 2,5-dibromo-3-methyl-6-isopropylbenzoquinone; DPI, diphenyleneiodonium; Lin, lincomycin; Nf, norflurazone. Asterisk indicates significant differences between the transformants treated with salt only and transformants co-treated with salt and other chemicals (** *p* < 0.01). (**c**) Schematic diagram of the *NPR1* construct introduced into *pBI121* for transient expression. (**d**–**f**) GFP fluorescence localization in cellular compartments of mesophyll protoplasts after transient expression with (**d**) *pNPR1::GFP-NPR1*, (**e**) *pNPR1::NPR1(∆5′35aa)-GFP*, and (**f**) *p35S::NPR1-CFP* and *pNPR1:: NPR1(∆NLS)-GFP*. Upper panel: CLSM images of GFP fluorescence (green) and chlorophyll autofluorescence from the chloroplasts (red) are merged in the third column. The bright-field image is merged into the fourth column. Lower panel: GFP intensity was quantified in the chloroplasts (*n* = 30). The GFP intensity was quantified in the nucleus using Image J (*n* = 30). Asterisks indicate significant differences between the 0 h time point and each of the different time points (** *p* < 0.01). Data are expressed as the mean ± SD. Scale bars = 5 μm.

The biphasic ethylene production involves various ACC synthase (*ACS*) genes, including *NtACS4* in the early phase at 1–3 h and *NtACS1* in the late phase at 48–72 h after pathogen infection [16]. To investigate the effect of ethylene production on NPR1 localization, we used *NtACS4*- and *NtACS1*-silenced transgenic plants (*NtACS4i* and *NtACS1i*, respectively) via RNA interference-mediated repression [16]. Stress-induced nuclear localization of NPR1-GFP was significantly reduced in protoplasts of both *NtACS4i* and *NtACS1i* transgenic plants (Figure 2a). Therefore, previous experiments have confirmed that nuclear NPR1 accumulation may be enhanced by stress-induced ethylene production.

Next, since the abundance of chloroplast NPR1 depends on H_2_O_2_ treatment, we examined whether altered chloroplast redox conditions affect nuclear NPR1 accumulation. In a previous study, we reported that under salt stress, plant cells rapidly upregulate the redox-sensitive NPR1 protein, which is imported into chloroplasts to induce protective responses against ROS accumulation, such as chaperones and antioxidants [7]. Specifically, we used 2,5-dibromo-3-methyl-6-isopropylbenzoquinone (DBMIB) to inhibit the photosynthetic electron transport chain in PS II [17] and increase the pool of reduced plastoquinone. However, in our current study, co-treatment with DBMIB and salt stress did not show any significant effect on nuclear NPR1 accumulation compared to salt stress alone (Figure 2b).

Subsequently, diphenyleneiodonium (DPI), an NADPH oxidase inhibitor [18], was administered to the mesophyll protoplasts of *pNPR1::NPR1-GFP* transgenic plants under salt stress. As expected, DPI treatment completely prevented nuclear NPR1 accumulation in the nucleus (Figure 2b), indicating that DPI-induced ROS generation significantly inhibited nuclear NPR1 accumulation. Norflurazon, a compound that generates ROS and causes photo-oxidative damage [19], induced a prominent nuclear NPR1 accumulation in leaf protoplasts. These results indicated that increased ROS production enhanced nuclear NPR1 accumulation. Collectively, these findings suggest that changes in the oxidative status of chloroplasts may affect nuclear NPR1 accumulation under stress. Additionally, lincomycin (Lin), a translation inhibitor in chloroplasts [20], significantly reduced nuclear NPR1 levels. Therefore, we next investigated the effect of translation in chloroplast on intracellular NPR1 localization.

We generated GFP- or CFP-fused constructs with various mutants, and investigated their transient expression in mesophyll protoplasts (Figure 2c). Despite GFP being present at the N-terminus of NPR1, GFP-NPR1 fluorescence was strongly detected in the chloroplasts under salt stress, exhibiting the same pattern as N-terminal tagging with GFP (*pNPR1::NPR1-GFP*) (Figure 2d and Figure S4). Additionally, N-terminal truncation, in which 35 amino acids were removed from the N-terminus of NPR1 (*pNPR1:: NPR1(∆5′35aa)-GFP*), exhibited strong fluorescence in the chloroplasts (Figure 2e and Figure S5). However, NPR1 fluorescence was not detected in the nuclei for either transient expression. These results indicate that the NPR1 is imported into the chloroplasts independently of its N-terminus.

NPR1 is controlled by the NLS at the C-terminus, and functions as a transcriptional coactivator in the nucleus [21]. We deleted the NLS region at the C-terminus of the NPR1, and the mutated construct, *pNPR1::NPR1(Δnls)-GFP*, was transiently co-expressed with *p35S::NPR1-CFP* in mesophyll protoplasts isolated from WT tobacco (Figure 2f and Figure S6). NPR1-CFP fluorescence intensity in the chloroplasts peaked at 6 h and gradually decreased thereafter. However, chloroplast NPR1-GFP levels in the *pNPR1::NPR1(Δnls)–GFP* transient expression were much higher than those in the *p35S::NPR1-CFP* transient protoplasts. NPR1(Δnls)-GFP fluorescence intensity in the chloroplasts continuously increased until 36 h. These results suggest that stress-induced NPR1 export from chloroplasts requires an NLS.

**Figure 3:**
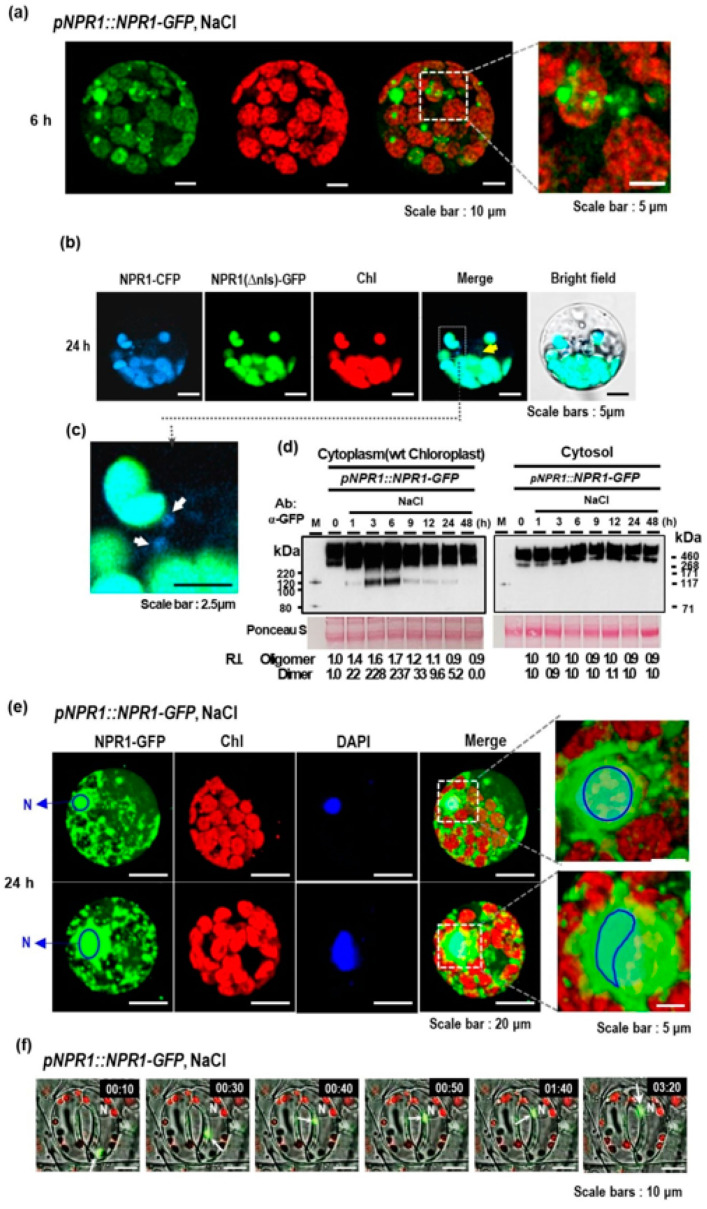
Intracellular localization of GFP-NPR1 in leaf protoplasts under salt stress. (**a**) CLSM images of GFP fluorescence in mesophyll protoplasts from 6-week-old *pNPR1::NPR1-GFP* transgenic plants exposed to salt stress using 200 mM NaCl. Images of GFP fluorescence (green) and chlorophyll autofluorescence from chloroplasts (red) are merged in the third column. Cytoplasmic vesicles containing NPR1-GFP were observed in the cytoplasmic region. NPR1-GFP is present within the green vesicles of various sizes. Cytoplasmic vesicles within the area enclosed by the dotted line are highlighted. (**b**,**c**) CLSM images following co-transient *pNPR1*-driven *GFP*-tagged nuclear localization sequence (NLS)-deleted *NPR1* (*p35S::NPR1(∆NLS)-GFP*) expression compared with *p35S*-driven NPR1-CFP expression in mesophyll protoplasts of WT under salt stress. The dotted white box in the merged column shows an image of vesicle-shaped NPR1-CFP protruding from the chloroplast (enlarged (**c**)). Nucleus (yellow arrow) and protrusions (white arrow). Images of (green) GFP fluorescence and (magenta) chlorophyll autofluorescence are merged in the fourth column. (**d**) Western blotting of subcellular protein fractions with anti-GFP antibody. The chloroplasts were first removed from the leaf cells of the transgenic plants, and then the supernatant was collected via centrifugation. Next, the cytoplasmic protein fraction in the pellet (sediment fraction) was harvested. Both protein fractions were used for Western blotting. Relative intensity was calculated by normalizing the band intensity values to the 0 h treatment, which was assigned as 1.0. (**e**) NPR1–GFP localization in the nucleus 24 h after salt stress. Highlights of the nuclear NPR1-GFP, merged with autofluorescence and DAPI images. In the enlarged image, the nuclear area within the dotted line is highlighted. (**f**) Snapshots of the rapidly moving cytoplasmic vesicles containing NPR1-GFP in the guard cells from the abaxial epidermis of *pNPR1::NPR1-GFP* transgenic plants 6 h after salt stress. White arrows indicate vesicles. N: nucleus.

Next, we investigated the mechanism of how the chloroplast NPR1 translocates into the nucleus. NPR1-GFP vesicles of various sizes were observed in the cytoplasm 6 h after salt stress (Figure 3a). Two constructs, *pNPR1::NPR1(Δnls)-GFP* and *p35S::NPR1-CFP*, were transiently co-expressed in mesophyll protoplasts isolated from WT tobacco leaves (Figure 3b,c). After salt stress, only NPR1-CFP was detected in the nucleus. Notably, vesicles containing only NPR1–CFP were detected around the chloroplasts, resembling chloroplast protrusions (Figure 3c).

To investigate the NPR1 migration pathway under high salinity conditions, chloroplasts and nuclei were isolated from plant leaves, and the cytoplasm was separated into liquid and sediment fractions, which were then subjected to Western blotting. The results showed that NPR1 was present in both the liquid and sediment fractions. While there was no significant change in NPR1 levels in the cytosol (liquid) fraction throughout the experiment, NPR1 levels in the sediment fraction without cytosol increased 3–6 h after exposure to stress. Notably, the levels of the NPR1 dimer and low-molecular-weight proteins increased in this fraction (Figure 3d). These findings suggest that NPR1 migration from the chloroplasts to the nucleus may occur through vesicles or other intracellular organelles via the cytoplasm.

The study further investigated the movement of vesicles containing NPR1-GFP molecules around intact chloroplasts and in the perinuclear region of the protoplasts in salt-stressed mesophyll cells (Figure 3e), indicating the intracellular trafficking of NPR1-GFP toward the nucleus. In mesophyll cells exposed to salt stress, the rapidly moving vesicles emitting GFP fluorescence were observed (Movie S1). After 24 h of salt stress, NPR1 was predominantly localized in and around the nucleus. The merged images of the blue nuclear fluorescence from 4′,6-diamidino-2-phenylindole (DAPI) staining and green fluorescent NPR1 signal showed a distinct blue-green signal, indicating NPR1 localization to the nucleus. The distinct green area surrounding the blue-green region indicated NPR1 localization to the perinuclear region, while the yellow region was visible outside the green area, suggesting the presence of NPR1-GFP inside the chloroplasts (Figure 3e, lower panel). It is interesting to note that abundant clusters of NPR1-GFP were observed around the nucleus. Based on the temporal changes in subcellular localization, the perinuclear aggregation of NPR1-GFP suggests that NPR1 exits the chloroplasts and moves toward the nucleus during the stress response. Therefore, these observations support the idea that NPR1 movement is linked to retrograde trafficking from the chloroplasts to the nucleus.

To further investigate whether NPR1 translocates from the chloroplasts to the nucleus more closely, the movement of the NPR1-GFP protein was examined in the guard cells of *pNPR1::NPR1-GFP* transformants using fluorescence microscopy (Thunder Imager, LEICA, Germany). Surprisingly, the NPR1 vesicles approaching the nucleus were observed to fuse with the nucleus, leading to a rapid disappearance of NPR1-GFP fluorescence, indicating that the NPR1 protein was imported into the nucleus (Figure 3f; Movie S2).

Two ankyrin proteins, STT1 and STT2, can form an intra-chloroplast liquid droplet cargo (thylakoid translocon) through the mechanism of liquid–liquid phase separation [22] and positively charged residues within the ankyrin domain are essential for oligomerization. The STT complex is mobile as an irregular aggregate, and NPR1-GFP mostly exists as oligomers in the cytoplasm. These findings suggest that during stress responses, NPR1 oligomers are released as vesicle-like structures or droplets from the chloroplast and directly access the nucleus.

Strong fluorescence and similar-sized proteins of NPR1-GFP were observed in the nuclei of tobacco mesophyll cells from *pNPR1::NPR1-GFP* and *p35S::cTP-NPR1-GFP* transformants under salt stress (Figure 1j and Figure S3a), as well as H_2_O_2_ or ACC application (Figure 1h and Figure S3b). These results reinforce that nuclear NPR1 accumulation through translocation from the chloroplasts depends on the ROS levels of the chloroplasts during the early stages of stress, and thus through the action of redox-sensitive chloroplast proteins. 

### 3.3. Changes in Nuclear NPR1 Accumulation According to Chloroplast Functionality

Only a small portion of the total chloroplast proteome that lacks cTP is encoded by nuclear DNA and synthesized in cytosolic ribosomes, entering the internal chloroplast compartments via endoplasmic reticulum (ER)-dependent pathways [23,24]. Several proteins that evolved from ankyrin repeat domains can bind to chloroplasts and specifically deliver cargo proteins to the chloroplast outer membrane [22,25]. The tobacco NPR1 (NtNPR1) contains two protein–protein interaction domains, four ankyrin repeat domains, a BTB domain with a zinc-finger motif, and an SA-binding domain (Figure S7), all of which are highly conserved in *Arabidopsis* NPR1 [26].

Although NtNPR1 lacks cTP and signal peptides, NPR1-GFP fluorescence, but not that of GFP alone, was observed in the chloroplasts, as evidenced by the yellow signal in the merged image with chloroplast autofluorescence (Figure S1). NPR3 (At5g45110) and NPR4 (At4g19660) were identified as nuclear-encoded chloroplast proteins in the Chloroplast Function Database II (http://rarge-v2.psc.riken.jp/chloroplast/, accessed on 2 February 2023), which provides information on Arabidopsis mutants of nuclear-encoded chloroplast proteins [27]. In particular, *npr3* mutants exhibit a pale green color phenotype. Phylogenetic analysis revealed that NPR3 and NPR4 are NPR1 paralogs that act as SA receptors [28] and evolved from an NPR ancestor (Figure S8). In homology modeling using SWISS-MODEL, NtNPR1 achieved a relatively high QMEANDisCo global score, even though its sequence shared only 40–50% identity with the AtNPR1 amino acid sequence, showing high confidence in almost the entire protein region (Figure S9). 

**Figure 4:**
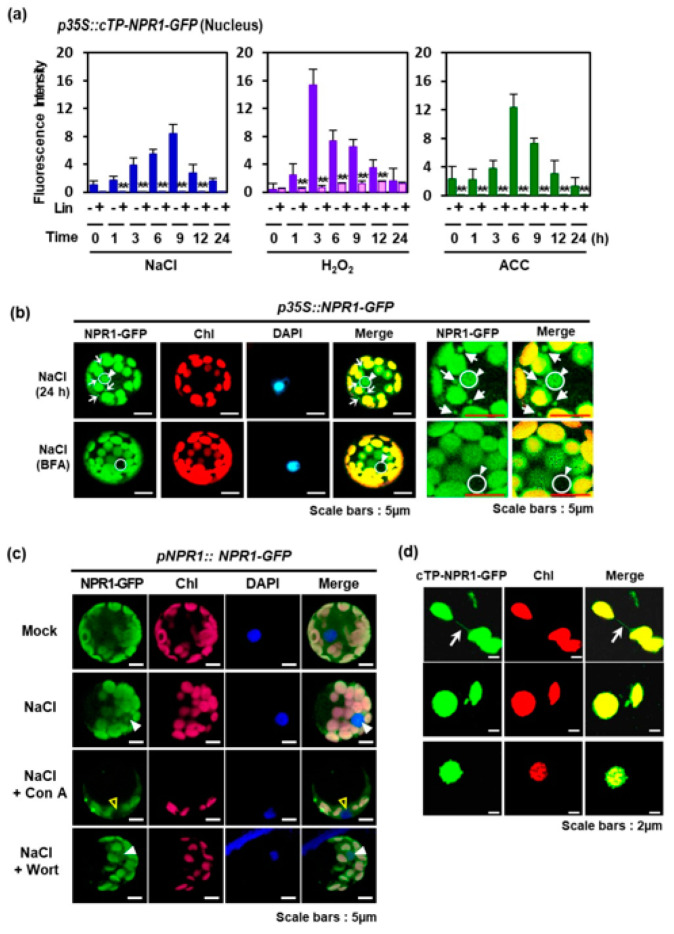
Regulatory factors affecting nuclear NPR1 accumulation. (**a**) GFP fluorescence intensity in the nucleus of mesophyll protoplasts from *p35S::cTP-NPR1-GFP* transgenic plants following salt stress, H_2_O_2_, or ACC application was visualized and quantified using Image J. GFP intensity in the nucleus of protoplasts was quantified after co-treatment with lincomycin (Lin) and NaCl, H_2_O_2_, or ACC (*n* = 30). Data are expressed as the mean ± SD. Asterisks indicate significant differences between Lin-treated and untreated cases (** *p* < 0.01). (**b**,**c**) CLSM images of NPR1-GFP fluorescence in salt-stressed protoplasts treated with (**b**) brefeldin A (BFA, bottom) and (**c**) concanamycin A and wortmannin. Fifth and sixth column (**b**): enlarged CSLM images. White circles and triangles indicate the whole nucleus. White arrows indicate cytoplasmic vesicles. (**d**) CLSM images of stromules (upper row), cytoplasmic vesicles (middle row), and chloroplast protrusions (lower row) in chloroplasts isolated from *p35S::cTP-NPR1-GFP* transgenic plants under salt stress. White arrows and triangles indicate stromules and chloroplast protrusions, respectively.

In particular, Lin markedly blocked NPR1 accumulation in the nucleus, regardless of H_2_O_2_ or ACC treatment under salt stress (Figure 4a). Lin, a prokaryotic translation inhibitor, blocks translation in the chloroplast without affecting cytoplasmic protein synthesis [20]. The retrograde signaling pathway was disrupted in Lin-treated plants [29]. These results suggest that NPR1 translocation from the chloroplast to the nucleus requires chloroplast function through protein translation, and the chloroplast-localized NPR1 migrates to the nucleus through the mediation of newly synthesized proteins. In conclusion, NPR1 is a suitable candidate for transmitting signals from the chloroplast to the nucleus through the cytoplasm.

To further investigate the role of chloroplasts in NPR1 translocation to the nucleus, subcellular NPR1 localization was examined in tobacco leaf epidermal pavement cells with small chloroplasts [30]. After salt stress, native *pNPR1*-driven NPR1-GFP accumulated significantly in the plasma membrane, cytoplasm, and nucleus of transgenic tobacco leaf abaxial epidermal pavement cells (Figure S10). In particular, NPR1-GFP of various sizes were dispersed in the cytoplasm after 6 h, and clear NPR1-GFP fluorescence was observed after salt stress and SA treatment for 24 h. The presence of minute cytoplasmic bodies in tobacco leaf epidermal pavement cells is consistent with the results of a previous report, indicating the presence of NPR1-GFP in the cytoplasm and nucleus of *Arabidopsis* after 5 mM SA treatment for 2 h, using a transient expression assay [31]. These NPR1 bodies were designated SA-induced NPR1 condensates, which are enriched with defense- and stress-associated proteins and ubiquitination components for protein homeostasis.

The fungal toxin brefeldin A, which disrupts intracellular vesicle trafficking mediated by ER and Golgi [32], reduced the number of cytoplasmic vesicles and NPR1-GFP fluorescence in the nucleus under salt stress (Figure 4b). In contrast, concanamycin A, a vacuolar H(+)-ATPase inhibitor [33], caused the degradation of NPR1-GFP fluorescence and chlorophyll via autophagic vacuoles in the cytoplasm (Figure 4c), but nuclear NPR1-GFP was not observed. When treated with wortmannin, an inhibitor of autophagosome formation [34], NPR1 accumulated in the nucleus, albeit in slightly reduced amounts (Figure 4c). Our findings indicate that the nuclear translocation of chloroplast NPR1 is not mediated by autophagic vesicles containing damaged chloroplast components.

In salt-stressed *p35S::cTP-NPR1-GFP* tobacco transformant, narrow protrusions from the chloroplast bodies showed GFP fluorescence (Figure 4d). These narrow minute structures are stromules, which are stroma-containing tubules that emanate from the main chloroplast body in vivo [35]. Stromules emanate from plastids at varying frequencies depending on environmental conditions and cell types [36]. Proteins, ROS, and other molecules flow through the stromules, which may transport retrograde signaling molecules from the chloroplasts to the nucleus. Stromules may be a route of plastid-derived vesicles for signaling environmental stimuli or recycling chloroplast content. Stromule-derived vesicles may function as NPR1 vehicles during chloroplast retrograde signaling via plastid–nuclear complexes through the Golgi bodies, ER, and nuclear envelope [37].

### 3.4. Enhancement of the Chloroplast NPR1 Related to Plant Tolerance of Salt Stress

**Figure 5:**
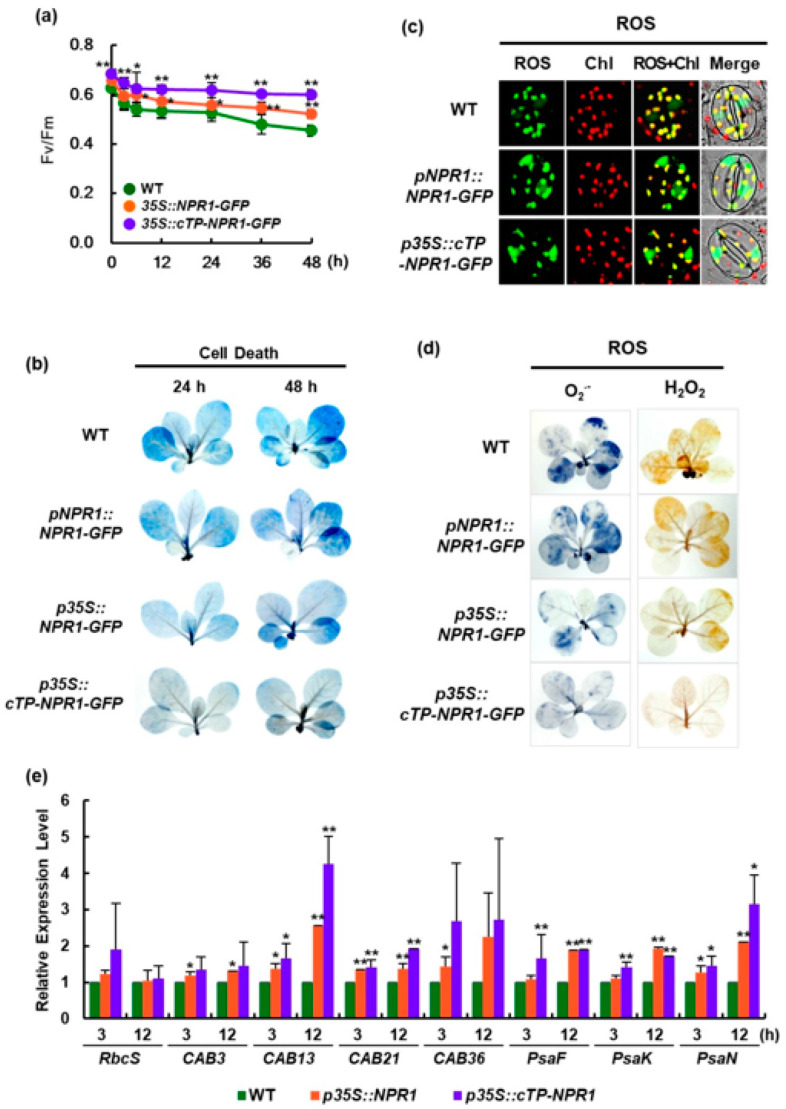
Enhancement of chloroplast-targeted NPR1 in stress resistance. (**a**) The maximal photochemical efficiency of photosystem II (*F*_v_/*F*_m_) was measured in WT, *p35S::NPR1-GFP*, and *p35S::cTP-NPR1-GFP* tobacco plants exposed to salt stress. Data are expressed as the mean ± SD. (**b**) Necrotic areas in salinity-stressed plants were stained with trypan blue. (**c**) Reactive oxygen species (ROS) accumulation in the compartments of guard cells in WT and transgenic plants 6 h under salt stress. ROS were visualized using CLSM after staining with 50 μM DCFH-DA. (**d**) Histochemical analysis of ROS accumulation. O_2_^−^ was detected with nitroblue tetrazolium staining (left), and H_2_O_2_ was detected with nitroblue tetrazolium staining (right). (**e**) Kinetics of nuclear-encoded gene transcription in WT and two transgenic plants under salt stress. Nuclear-encoded genes: *RbcS*, RuBisCo small subunit; *CAB3*, chlorophyll *a*/*b*-binding protein 3; *CAB12*, chlorophyll *a*/*b*-binding protein 12, *CAB21*, chlorophyll *a*/*b*-binding protein 21; *CAB36*, chlorophyll *a*/*b*-binding protein 36; *PsaF*, photosystem I reaction center subunit III; *PsaK*, photosystem I subunit X; *PsaN*, photosystem I reaction center subunit XII. Relative transcript expression levels are presented as mean ± SD. Significant difference between WT and transgenic plants at the indicated time points are shown as * *p* < 0.05 or ** *p* < 0.01.

Maximal photochemical efficiency and trypan blue staining for cell death indicated that *p35S*-driven *NPR1-GFP* overexpression increased tolerance to salt stress. Compared to WT, transgenic plants expressing *cTP-NPR1-GFP* or *NPR1-GFP* showed enhanced maximal photochemical efficiency of PSII (*F*_v_*/F*_m_) under salt stress, as evidenced by chlorophyll fluorescence measured using the PAM 2000 photosynthesis yield analyzer (Walz, Germany) (Figure 5a). Constitutive *cTP-NPR1-GFP* expression significantly reduced cell damage under salt stress compared to NPR1 without cTP (Figure 5b and Figure S11). However, salt--stress-mediated cell damage in transgenic plants with native *pNPR1*-induced NPR1 was not notably different from that in WT plants. These results can be explained by the functions of the chloroplast-localized NPR1 as an antioxidant and chaperone [7].

Guard cells initiate a rapid physical reaction used by land plants to alter their interactions with the environment. Several studies have demonstrated the interplay between Ca^2+^ and ROS in guard cell and their potential co-amplification effects [38]. At the cellular level, ROS accumulation was first investigated in the nuclei and chloroplasts of guard cells under salt stress using DCFH-DA. Although high levels of ROS were detected in the chloroplasts of WT, their levels were markedly lower and higher in the chloroplasts and nuclei of guard cells in both transgenic plants, respectively (Figure 5c).

Here, we visualized in vivo ROS generation using nitroblue tetrazolium and diaminobenzidine staining for histochemical detection of O_2_^·−^ and H_2_O_2_, respectively. Leaves with increased NPR1 expression showed decreased ROS accumulation in the chloroplast compared to controls under salt stress (Figure 5d). Particularly, the cTP-fused NPR1 further suppressed O_2_^·−^ and H_2_O_2_ accumulation in the leaves of transgenic plants under salt stress. These results suggest that elevated NPR1 content in chloroplasts most efficiently inhibited ROS generation in *p35S::cTP-NPR1-GFP* transformants.

In *p35S::cTP-NPR1-GFP* transgenic plants, the increase pattern in *F*_v_*/F*_m_ was consistent with the increased gene expression characteristics of chloroplast components for photosynthesis (Figure 5e). To elucidate the physiological functions of nuclear NPR1 in response to salt stress, the transcription patterns of nuclear-encoded genes for photosynthesis-related proteins were compared between WT and transgenic plants (*p35S::NPR1-GFP* and *p35S::cTP-NPR1-GFP*) under salt stress. Real-time quantitative RT-PCR (qRT-PCR) was performed on genes encoding RuBisCo, core complex, and PS I and II antenna proteins. At 3 and 12 h after salt stress, transcript levels of almost all genes were higher in transgenic plants than those in WT plants. In particular, compared to WT, the transcript levels of all tested nuclear-encoded genes in *p35S::cTP-NPR1-GFP* were significantly higher 12 h after salt stress.

**Figure 6:**
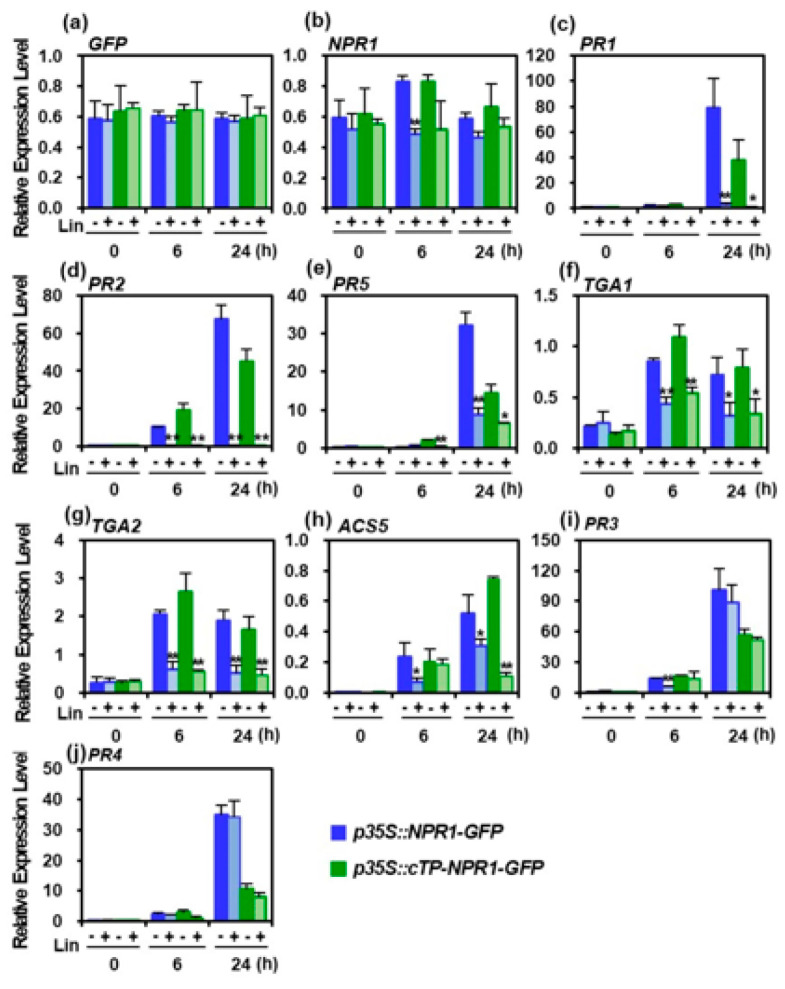
Transcription of pathogenesis-related (PR) and stress-related genes in the presence or the absence of lincomycin (Lin) treatment under salt stress. Transcription levels in response to 200 mM NaCl-induced salt stress in *p35S::NPR1-GFP* and *p35S::cTP-NPR1-GFP* tobacco plants, with or without Lin treatment, were measured using qRT-PCR and expressed relative to those of the reference gene *β-actin*. Relative transcript expression levels were presented as the mean ± SD. Asterisk indicates significant differences between Lin-treated and untreated cases (* *p* < 0.05 or ** *p* < 0.01). Data from five independent experiments with *n* = 3 are presented.

In plant immune responses, nuclear NPR1 interacts with transcription factors of the TGA class of basic domain/leucine zippers and induces the expression of pathogenesis-related (*PR*) gene [39]. To investigate whether nuclear NPR1 translocation from chloroplasts affects *PR* gene transcription under salt stress, *GFP* transcript levels in *p35S::cTP-NPR1-GFP* and *p35S::NPR1-GFP* transgenic plants with or without Lin treatment were examined using qRT-PCR. GFP transcription in both transformants, with or without Lin treatment, did not change, implying that *35S* promoter was not affected by NPR1 localization (Figure 6a). 

However, *NPR1* transcript levels increased in both transformants under salt stress only in the absence of Lin, which may be due to the stress-induced NPR1 transcript itself (Figure 6b). Surprisingly, the transcript levels of the SAR marker genes *PR1*, *PR2*, and *PR5* significantly increased after 24 h in Lin-untreated stressed transformants, which was abolished after Lin treatment (Figure 6c–e). Transcript levels of TGA members *TGA1* and *TGA2* significantly decreased in the presence of Lin (Figure 6f,g). Additionally, the transcript levels of *ACS5*, an ACC synthase involved in ethylene biosynthesis, decreased considerably following Lin treatment (Figure 6h). However, the expression levels of *PR3* and *PR4*, which are jasmonic-acid-induced genes, were not significantly different, regardless of the presence or absence of Lin for 24 h (Figure 6i,j). Overall, SA marker genes and TGA transcription factors were induced by nuclear NPR1, thereby attenuating photosynthetic loss, alleviating cell damage, and inducing stress tolerance. Therefore, NPR1 translocation from the chloroplast to the nucleus plays a physiological role in stress tolerance induction. 

### 3.5. NPR1 Involvement in Retrograde Signaling Communication

**Figure 7:**
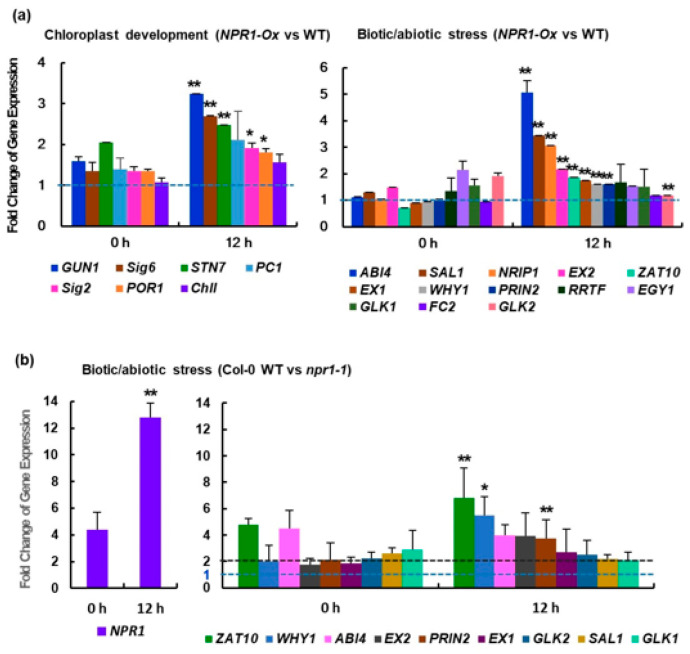
Expression analysis of retrograde-signaling-related genes. (**a**) Expression fold changes of retrograde-signaling-related genes involved in (left) chloroplast development and (right) biotic/abiotic stress response in NPR1 overexpression (*NPR1-Ox*) versus WT plants after salt stress (200 mM). (**b**) Expression fold changes of retrograde signaling-related genes involved in biotic/abiotic stress response in Col-0 WT versus *npr1-1* mutant plants after salt stress (100 mM). The expression fold change was calculated based on the relative expression level of each gene in WT versus (**a**) *NPR1-Ox* or (**b**) *npr1-1* mutant after salt stress. Chloroplast development: *GUN1*, genomes uncoupled 1; *Sig6*, chloroplast sigma factor 6; *STN7*, serine/threonine-protein kinase 7; *Sig2*, chloroplast sigma factor 2; *NADPH:POR1*, protochlorophyllide oxidoreductase; *PC1*, plastocyanin; *ChlI*, magnesium–protoporyphyrin chelatase subunit. Biotic/abiotic stress: *ABI4*, abscisic acid-insensitive protein 4; *SAL1*, 3′-phosphoadenosine 5′-phosphate phosphatase; *NRIP1*, N-receptor-interacting protein; *EX2*, executer 2; *GLK1*, golden 2-like 1; *ZAT10*, zinc-finger transcription factor 10; *EX1*, executer 1; *WHY1*, single-stranded DNA-binding protein WHIRLY 1; *PRIN2*, plastid redox insensitive 2; *EGY1*, ethylene-dependent gravitropism-deficient and yellow-green 1; *RRTF*, redox-responsive transcription factor; *FC2*, ferrochelatase; *GLK2*, golden 2-like 2; *NPR1*, non-expressor of pathogenesis-related gene 1. Data are expressed as the mean ± SD. The dashed lines indicate 1-fold and 2-fold changes in expression, respectively, in *NPR1-Ox versus* WT of tobacco (**a**) and Col-0 WT versus *npr1-1* mutant of *Arabidopsis* (**b**). Asterisks indicate significant differences between the following pairs: tobacco WT and *NPR1-Ox*, as well as *Arabidopsis* Col-0 WT and *npr1-1*, at the indicated time points (* *p* < 0.05 or ** *p* < 0.01).

While many retrograde signaling proteins remain elusive, several plastid proteins, including NB-LRR receptor-interacting protein 1 (NRIP1), EXECUTER1 (EX1), EX2, GENOMES UNCOUPLED1 (GUN1), and the single-stranded DNA-binding protein, WHIRLY 1, have been implicated in chloroplast retrograde signaling [3,40,41]. Molecules involved in retrograde signaling communication in plants are divided into two groups: chloroplast biogenesis and development components, and stress response and immunity components. We determined the transcriptome level changes of retrograde-signaling-related proteins expressed in *NPR1-Ox* lines compared to WT. Under high salt conditions, the levels of nuclear transcripts of retrograde-signaling-related proteins involved in chloroplast development were higher in *NPR1-Ox* lines than that in WT plants. Real-time RT-PCR was used to determine this, and it was more pronounced at 12 h than at 1 h (Figure 7a and Figure S12a). In particular, the transcripts of sigma factors (SIG) *sig2* and *sig6*, as well as *GUN1* and *STN7*, were markedly increased. Sigma factors are important retrograde signaling proteins that activate plastid-encoded RNA polymerase [42].

Furthermore, the transcript levels of nuclear-encoded genes involved in stress-related retrograde signaling were studied under salt stress conditions. These transcripts showed stress-induced patterns, with higher transcript levels in *NPR1-Ox* plants than those in WT plants (Figure 7a, right). The transcript levels were the highest 12 h after initiating the salt stress, increasing transiently (Figure S12b). Several operational retrograde signals, such as SAL1, NRIP1, EX1, EX2, ZAT10, and WHY1, which are encoded by nuclear transcripts, may play a role in activating adaptive responses to environmental changes. This pattern is consistent with the nuclear NPR1 localization pattern under salt stress. Although the transcription factor ABI4 was previously shown to not play a role in biogenic chloroplast-to-nucleus retrograde signaling [41], its level was more increased in the *NPR1-Ox* transformants than that in the WT.

To further investigate the physiological functions of NPR1 in response to salt stress, we compared Col-0 WT and *npr1-1* mutant *Arabidopsis* plants. *F*_v_*/F*_m_ was reduced in the *npr1-1* mutant under salt stress compared to the WT (Figure S13a), which was consistent with the gene expression characteristics of several chloroplast components involved in photosynthesis (Figure S13b). Salt stress significantly decreased the expression levels of photosynthesis-related genes encoded in chloroplasts, although the decrease was more severe in the *npr1-1* mutant, implying that the NPR1 positively regulates gene expression for photosynthesis in chloroplasts.

To investigate whether defective NPR1 affects the expression of retrograde communication-associated nuclear genes, we performed real-time RT-PCR. Under high salinity, the expression levels of almost all analyzed genes remained similar to the basal level in the *npr1-1* mutant during the entire period of salt stress, strongly contrasting with the gene expression pattern in Col-0 WT plants (Figure S14). The transcript levels of all analyzed genes involved in the chloroplast–nuclear retrograde signaling network were significantly higher in Col-0 WT plants than those in *npr1-1* mutants (Figure 7b). In particular, transcripts of the transcription factors ABI4 and ZAT10, which are involved in ABA and jasmonic acid signaling, respectively, increased significantly. During the entire period of salt stress treatment, the transcript levels of retrograde signaling-related genes, including *ZAT10*, *WHY1*, *EX1*, *EX2*, *PRIN2*, *GLK1*, *GLK2*, and *SAL1* [3,41], were significantly higher in the Col-0 WT than that in the *npr1-1* mutants. This suggests that the NPR1 plays a crucial role in regulating the retrograde signaling pathway.

## 4. Discussion

Chloroplasts have been found to function as sensors of environmental and developmental cues, influencing photosynthesis and transmitting information to the nucleus to coordinate plant growth, development, and stress responses [3,43,44]. Certain dual-target proteins, which are capable of targeting both the nucleus and other cell organelles, are believed to play a role in signal transduction pathways [45]. One such protein, WHIRLY1, was the first to be identified in both the chloroplasts and nucleus of the same cells, and has been shown to be involved in maintaining plastid genome stability by interacting with nucleoids [46,47]. WHIRLY1, which is associated with the thylakoid membrane, can also act as a redox sensor for retrograde signaling from the chloroplast to the nucleus [48]. Additionally, it has been proposed that EX1 is directly involved in mediating retrograde singlet oxygen signaling from the plastid to the nucleus [49].

Retrograde signaling regulates nuclear gene expression in response to developmental status, abiotic or biotic stresses, and innate immunity [50]. Recent studies have suggested several chloroplast retrograde signals, including carotenoid derivatives, isoprenoid precursors (methylerythritol cyclodiphosphate), 3ʹ-phosphoadenosine 5ʹ-phosphate, tetrapyrroles, heme, ROS, and transcription factors [3,51]. These signals and related pathways form a communication network that regulates nuclear gene expression, miRNA biogenesis, RNA editing, and gene splicing to help plants adapt to developmental and stress stimuli [50,52,53]. Despite the identification of several retrograde signaling modules, our understanding of the regulatory complexity in these pathways remains limited. Since retrograde signaling regulation has been partially explained, little is known about how these signals are transmitted to the nucleus. Here, it is suggested that NPR1 may acts as a mobile macromolecule that translocates from the chloroplasts to the nucleus.

In this study, we observed dual NPR1exhibits in both the chloroplasts and the nucleus when exposed to high salt stress and stress-signaling molecules (Figure 1). This dual localization of NPR1 may indicate a role in retrograde signaling to sense environmental changes and trigger acclimatory responses that affect nuclear gene expression. While the chloroplast NPR1 level remained relatively stable under unstressed conditions and was only slightly elevated by salt stress, the nuclear NPR1 level was transiently induced at specific times during stress conditions. This indicates that the level of NPR1 in the nucleus reflects the environmental signals from the chloroplasts through the retrograde signaling network. 

Our previous findings have shown that NPR1 exhibits functional differences in chloroplasts and the nucleus. In chloroplasts, NPR1 exhibits molecular chaperone activity for emergency restoration associated with proteostasis and redox homeostasis, whereas in the nucleus, it functions as a transcriptional coactivator for stress adaptation [7]. Although most dual nuclear–chloroplast-localized proteins may be related to regulatory storage in chloroplasts, their molecular functions appear to be compartment-specific [45].

In immunoblot assays and GFP fluorescence imaging, we found that the amount of NPR1-GFP in the nucleus was greatly enhanced at 12 h, indicating an increased influx of NPR1 into the nucleus (Figure 1). Experiments with NPR1-GFP and GFP-NPR1, in which the order of the N-terminus was altered, or with ∆5′35aaNPR1-GFP, in which 35 amino acids were partially deleted from the 5′ end of NPR1, completely prevented nuclear NPR1 accumulation. Instead, the level of NPR1 increased in the chloroplast. Furthermore, the deletion of the NLS from the NPR1 protein has led to inefficient nuclear localization, resulting in NPR1 overaccumulation in the chloroplast and blocking the chloroplast-to-nucleus retrograde signaling (Figure 2).

In addition, the inhibition of protein synthesis in the chloroplasts via Lin treatment significantly reduced the NPR1 level in the nucleus (Figure 2b and Figure 4a), which was accompanied by a decrease in the transcripts of SAR-related genes activated by the transcriptional coactivator NPR1 (Figure 6). Notably, the transcript levels of PR1, a marker gene for SA-mediated signaling, were prominently suppressed by Lin treatment. Similarly, Lin treatment led to a significant reduction in the transcript levels of *PR2*, *PR5*, *TGA1*, and *TGA2*, as NPR1’s regulatory role as a transcriptional coactivator in the nucleus was blocked [13,39]. However, the transcript levels of *PR3* and *PR4*, which are marker genes regulated by jasmonic acid, were unchanged, because NPR1 was not involved in their expression. Therefore, these findings imply that retrograde movement of NPR1 from chloroplasts to the nucleus is necessary for the expression of SAR. Hence, NPR1-mediated nuclear gene expression is responsible for NPR1 retrograde signaling from the chloroplasts to the nucleus, which contributes to stress tolerance. Furthermore, this retrograde signaling induces the resultant transcriptional changes that attenuate the loss of photosynthetic capability, alleviates cell damage, and enhances stress tolerance.

The redox state of the photosynthetic electron transport chain may trigger the movement of WHIRLY1 from the chloroplasts to the nucleus, similar to the regulation of NPR1 translocation from the cytosol to the nucleus [48]. Moreover, WHIRLY1 has been shown to promote SA synthesis, suggesting that NPR1 may be a part of retrograde signaling through its translocation [54]. In this study, the expression of genes associated with well-known retrograde signaling components was transiently increased in *NPR1-Ox* compared to the tobacco WT, whereas their expression in the *Arabidopsis npr1-1* mutant remained at the basal level (Figure 7). These results indicate that NPR1 is directly associated with retrograde signaling pathways that are involved in nuclear gene regulation mediated by SA.

Experiments with the cTP-NPR1-GFP protein confirmed that NPR1 accumulates in the nucleus after possibly transitioning through the chloroplast after cTP cleavage (Figure 1). The monomeric NPR1-GFP protein, with a size of 92.3 kDa, was detected in the nuclear protein fraction of *pNPR1::cTP-NPR1-GFP* transformants after the application of NaCl, H_2_O_2_, or ACC (Figure 1). The detection of the 92.3 kDa protein was due to the removal of the cTP from the N-terminus of cTP-NPR1-GFP by the stromal processing peptidase [15]. In both *pNPR1::cTP-NPR1-GFP* and *pNPR1::NPR1-GFP* transgenic plants, NPR1-GFP monomers of the same size were detected in the nucleus, indicating that the NPR1 was transferred from the chloroplast to the nucleus. This process suggests that the previously entered NPR1 in the chloroplast, senses redox changes such as chloroplast-derived ROS, and conveys the information to the nucleus to regulate gene expression through subsequent NPR1 translocation. NPR1 translocation to the nucleus might occur through several mechanisms, potentially involving unknown proteins, stromules, or vesicle-mediated transport (Figure 3 and Figure 4).

Recently, signaling through mobile transcription factors has been recognized as a communication route between cell organelles and the nucleus. Transcription factors, including chloroplast import appratus2 (CIA2), CIA2-like (CIL), WHIRLY, and plant homeodomain (PHD), exhibit dual nuclear–plastid localization, suggesting their possible involvement in retrograde signaling [45]. In *Arabidopsis*, CIA2 and CIL are involved in regulating responses to UV-AB, high light, and heat stress through chloroplast–nucleus crosstalk, for instance, by regulating thermotolerance through heat shock protein expression.

Nonetheless, the exact regulatory mechanism underlying the mediation of plastid-to-nucleus retrograde signaling remains unknown. NPR1 and WHIRLY1 exhibit similarities in that both proteins display dual functions and localization in the chloroplasts and nucleus. The possibility that NPR1 and WHIRL1 exist as two isoforms cannot be completely ruled out, given their compartment-specific molecular functions [55].

## 5. Conclusions

Our study aimed to investigate the functional significance of the chloroplast NPR1 and its role in retrograde signaling, which plays a critical role in regulating proper allocation under stress conditions. We explored the intracellular translocation of NPR1 as a potential physiological strategy for functional switching between dual localizations. Retrograde NPR1 signaling, which is triggered by redox status or stress-induced components in chloroplasts, is a crucial environmental stress regulatory mechanism in plants. Chloroplast-to-nucleus retrograde signaling is essential for adaptable plant growth and development. Our findings suggest that the chloroplast NPR1 may serve as a retrograde communicator for the protective machinery from the chloroplasts to the nucleus in a redox-mediated manner, particularly in photosynthetically active leaf cells under adverse environmental conditions.

## Data Availability

All data are available in the manuscript or Supplementary Materials. The tobacco NPR1 sequence was deposited in the GenBank database under accession number KY402167. Some of the data can be found in our preprint paper (bioRxiv preprint doi: https://doi.org/10.1101/2021.03.24.436779) on bioRxiv, which is a preprint server for biology.

## Supplementary Materials

The following supporting information can be downloaded at: https://www.mdpi.com/article/10.3390/antiox12051118/s1, Figure S1: Green fluorescent protein (GFP) fluorescence localization and immunoblot analysis in the cellular compartments of *p35S::GFP* transgenic plants under salt stress; Figure S2: Immunoblots of the total and chloroplast protein fractions from *pNPR1::NPR1-GFP* under salt stress; Figure S3: CLSM images of GFP fluorescence on mesophyll protoplasts after treatments with salt stress, H_2_O_2_ or ACC; Figure S4: NPR1-GFP localization in salt stress; Figure S5: Subcellular localization of *NPR1(∆5′35aa)*-GFP; Figure S6: Subcellular localization of nuclear localization signal (NLS)-deleted NPR1-GFP mutant; Figure S7: Alignment of nonexpressor of pathogenesis-related genes 1 (NPR1) proteins from several plants; Figure S8: Phylogenetic tree constructed using the neighbor-joining method in MEGA version 11.0 based on the sequences of 28 NPR1 genes from different plants species; Figure S9: Information of functional domains and homology model NPR1 protein from *Nicotiana tabacum* (NtNPR1, KY402167) for functional analysis; Figure S10: GFP fluorescence in the chloroplasts and nucleus of *p35S::cTP-NPR1-GFP* transgenic tobacco plants under salt stress; Figure S11: Phenotypes of WT and two transgenic tobacco lines under salt stress. Whole tobacco plants (WT, *pNPR1::NPR1-GFP*, and *p35S::cTP-NPR1-GFP*) were exposed to salt stress for 48 h; Figure S12: Transcription of retrograde-signaling-related genes involved in chloroplast development and abiotic/biotic stress response in wild-type (WT) and NPR1 overexpression (*NPR1-Ox*) transgenic tobacco plants under salt stress; Figure S13: Kinetics of maximal photochemical efficiency and chloroplast-encoded gene transcription in WT and *npr1-1* mutant *Arabidopsis* plants under salt stress; Figure S14: Transcription of retrograde-signaling-related genes in response to abiotic/biotic stress in WT and *npr1-1* mutant *Arabidopsis* plants under salt stress; Table S1: Sequences of primers for real-time qRT-PCR; Movie S1: Time-lapse movie of merged NPR1-GFP images with GFP (green), chlorophyll autofluorescence from chloroplasts (red), and blue staining of DAPI in mesophyll protoplasts of *pNPR1::NPR1-GFP* transgenic plants 24 h after salt stress; Movie S2: Time-lapse movie of fluoromicroscopy images for NPR1-GFP in guard cells of *pNPR1::NPR1-GFP* transgenic plants 6 h after salt stress; The full scan of the entire original blots: Figure 1a,b,d,g,j and Figure 3d.
