# Supplementary material for: NPR1 Translocation from Chloroplast to Nucleus Activates Plant Tolerance to Salt Stress
Source: Antioxidants (Basel). 2023 May 18;12(5):1118. doi: 10.3390/antiox12051118 (PMC10215103; doi:10.3390/antiox12051118)
Supplement: Supplementary file 1 [file antioxidants-12-01118-s001.zip › Supplementary Figures.pdf]

Figure S1

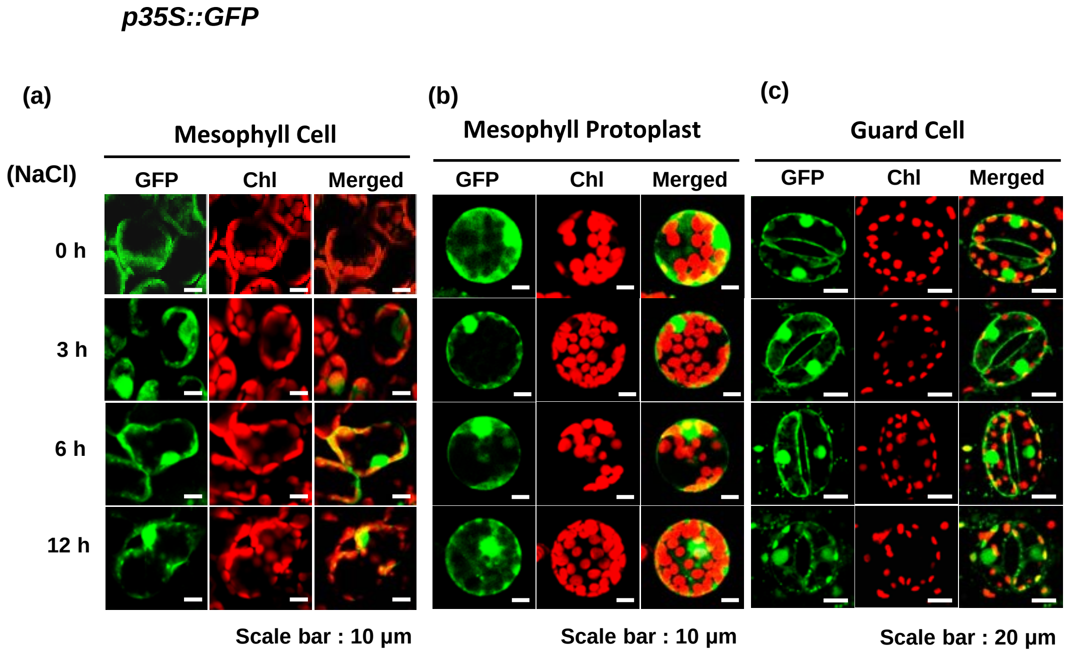

Figure S1. Green fluorescent protein (GFP) fluorescence localization and immunoblot analysis in the cellular compartments of *p35S::GFP* transgenic plants under salt stress. (**a–c**) Confocal laser scanning microscopy (CLSM) images of (green) GFP fluorescence were observed in (a) mesophyll cells, (b) mesophyll protoplasts, and (c) guard cells of 6-week-old plants exposed to salt stress with 200 mM NaCl. CLSM images of (green) GFP fluorescence and (red) chlorophyll autofluorescence from chloroplasts are merged in the third column.

Figure S2

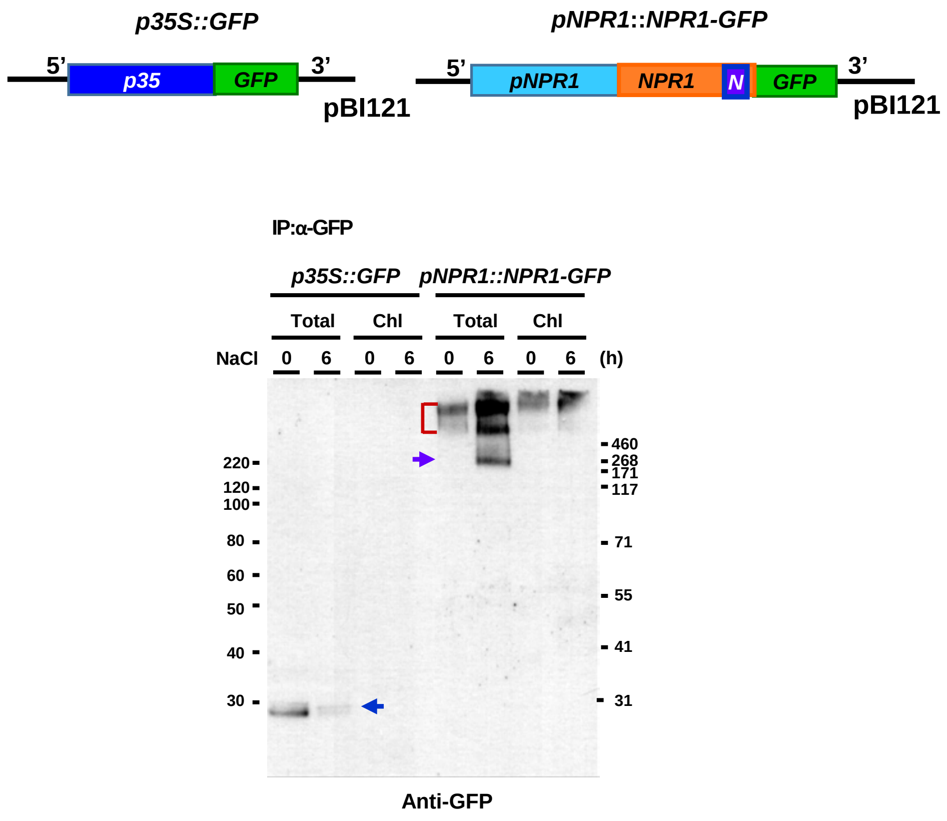

Figure S2. Immunoblots of the total and chloroplast protein fractions from *pNPR1::NPR1-GFP* under salt stress. After immunoprecipitating the protein fractions using GFP-Trap agarose, western blotting with anti-GFP antibody was performed. GFP-tagged nonexpressor of pathogenesis-related gene 1 (NPR1-GFP) oligomer (high molecular weight) and dimer (low molecular weight) are indicated with red square brackets. Purple arrow indicates GFP dimer. Blue arrow indicates GFP only in total protein fraction in *p35S::GFP* transformants.

Figure S3

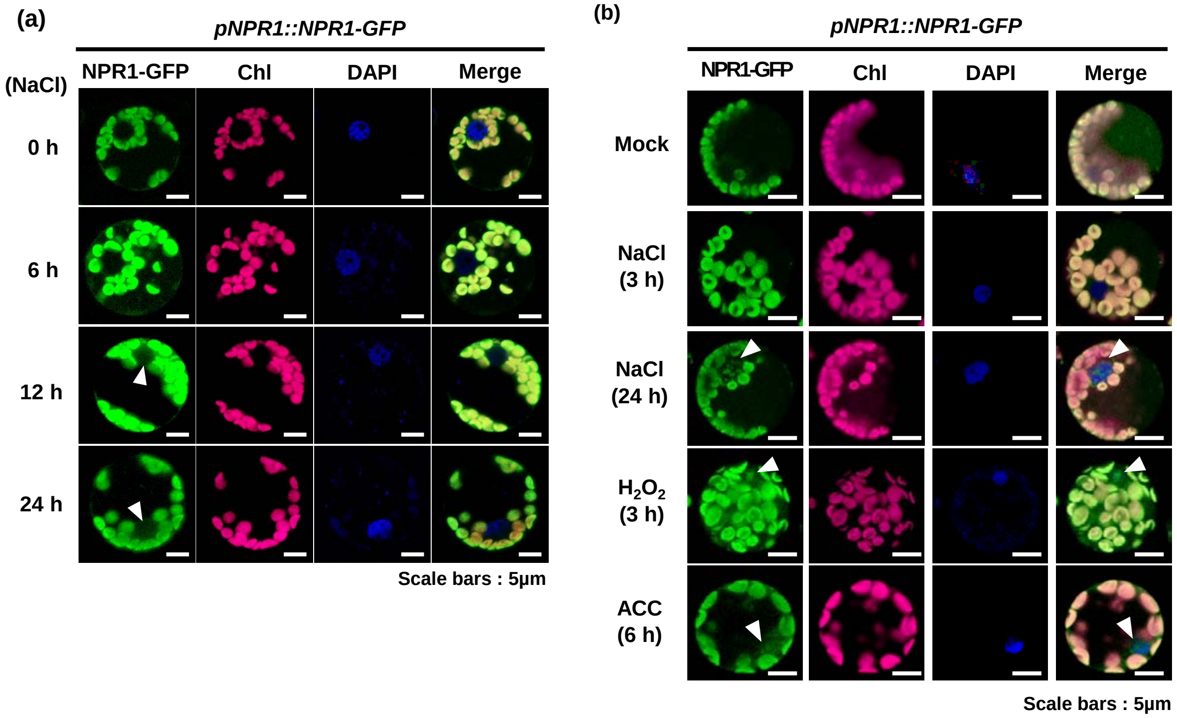

Figure S3. CLSM images of GFP fluorescence on mesophyll protoplasts after treatments with salt stress, H_2O_2 or ACC. **(a)** CLSM images of GFP fluorescence in mesophyll protoplasts from 6-week-old *pNPR1::NPR1-GFP* transgenic plants exposed to salt stress using 200 mM NaCl. (**b**) CLSM images of GFP fluorescence in mesophyll protoplasts from *pNPR1::NPR1-GFP* transgenic plants treated with 200 mM NaCl, 5 mM H_2O_2, or 1 mM 1-amino-1-cyclopropane carboxylic acid (ACC). Images of (green) GFP fluorescence and (magenta) chlorophyll autofluorescence from chloroplasts are merged in the fourth column. DAPI staining (blue) images are presented in the third column.

Figure S4

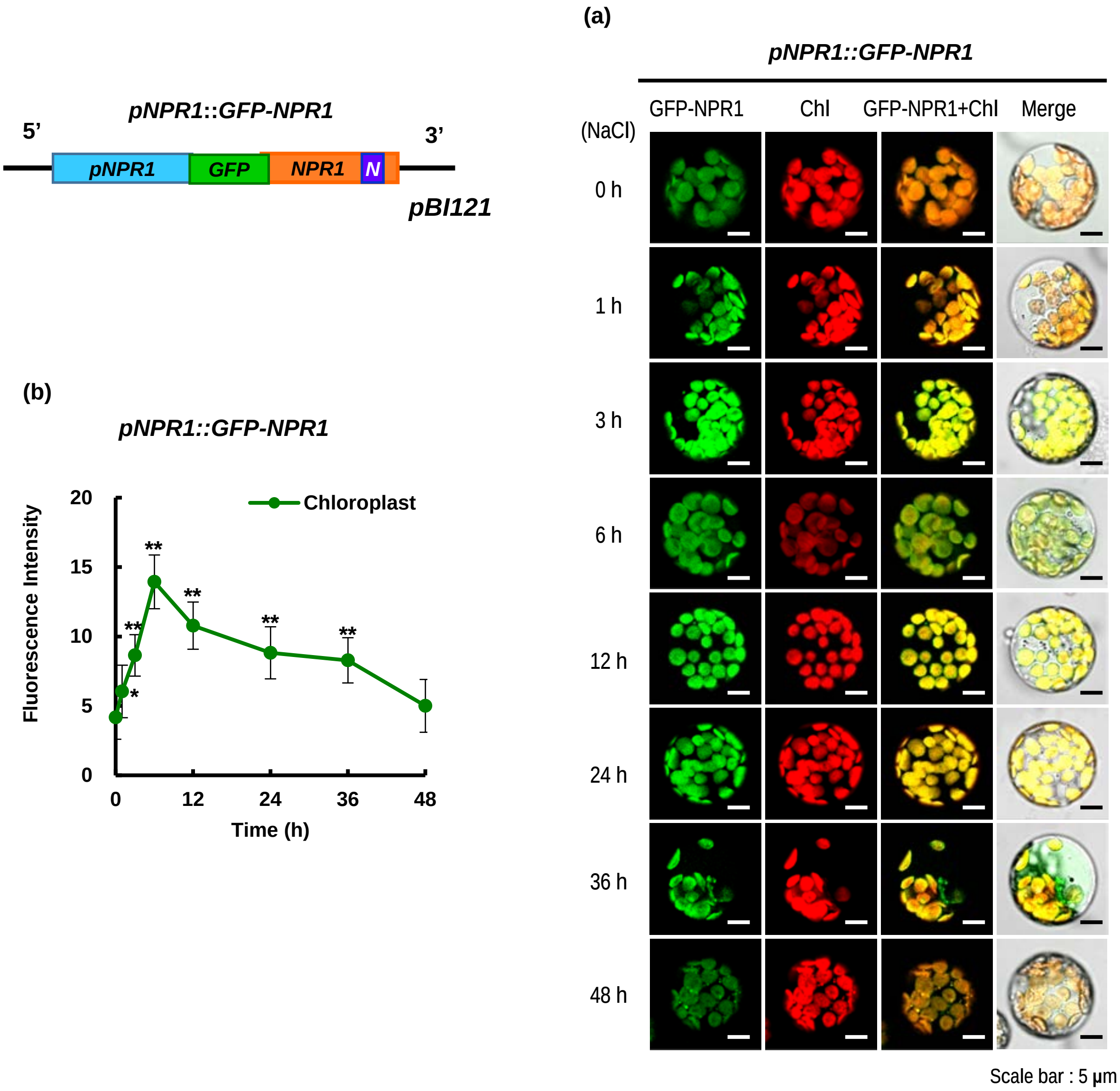

Figure S4. NPR1-GFP localization in salt stress. (**a**) GFP fluorescence localization in cellular compartments of mesophyll protoplasts after transient expression with *pNPR1::NPR1-GFP* construct. Confocal laser scanning microscopy (CLSM) images of (green) GFP fluorescence and (red) chlorophyll autofluorescence from chloroplasts are merged in the third column. The bright-field image is merged into the fourth column. (**b**) GFP intensity was quantified in the chloroplasts after transient *pNPR1::NPR1-GFP* (green line) expression under salt stress (n=30). Data are expressed as mean ± SD. An asterisk indicates a significant difference from 0 h (one asterisk (P<0.05) or two asterisks (P < 0.01)).

Figure S5

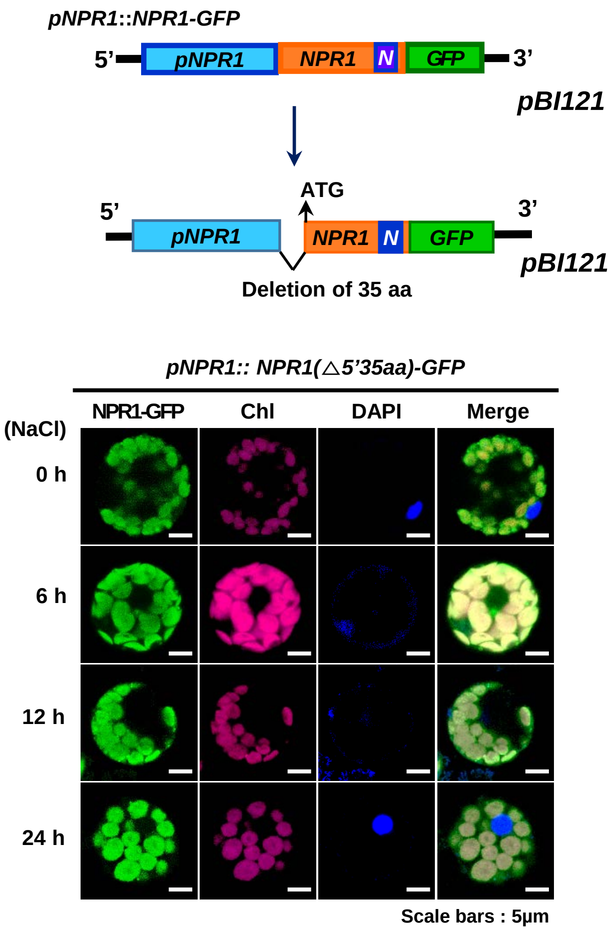

Figure S5. Subcellular localization of *NPR1(Δ5'35aa)*-GFP. GFP-tagged nonexpressor of pathogenesis-related genes 1 (GFP–NPR1) localization in intracellular compartments of mesophyll protoplasts after transient *pNPR1::NPR1(Δ5'35aa)-GFP* expression. After deleting the N-terminal 35 amino acids from the NPR1 open reading frame, a deletion construct with the ATG start codon was constructed. GFP fluorescence localization in mesophyll protoplasts after transient *pNPR1::NPR1(Δ5'35aa)-GFP* construct expression. Confocal laser scanning microscopy (CLSM) images of (green) GFP fluorescence and (magenta) chlorophyll autofluorescence from chloroplasts are merged in the third column. The bright-field image is merged into the fourth column. Scale bars = 5 μm.

Figure S6

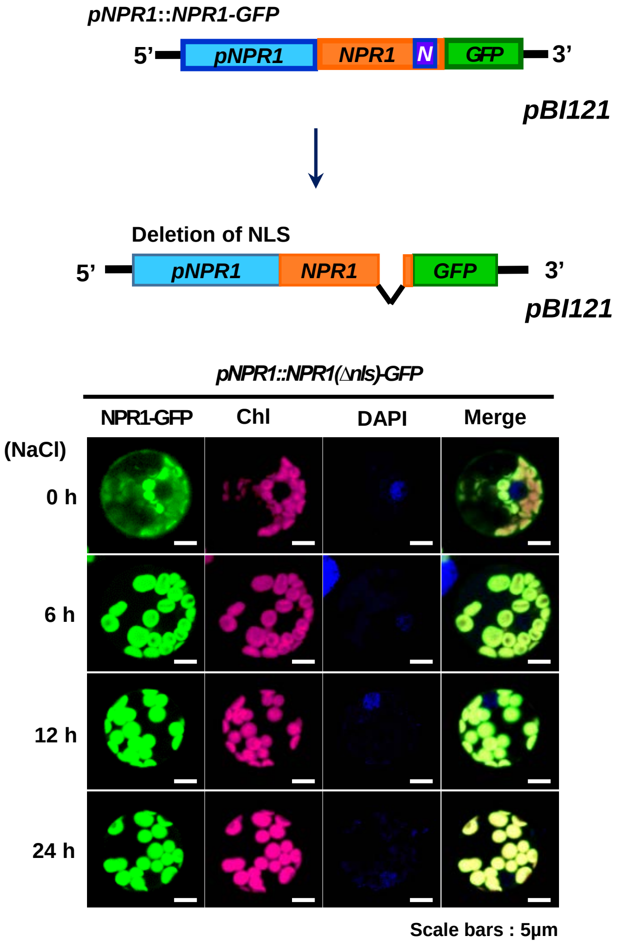

Figure S6. Subcellular localization of nuclear localization signal (NLS)-deleted NPR1-GFP mutant. (**a**) Deletion constructs of the NLS within the NPR1 open reading frame were subcloned in frame with a C-terminal GFP and under the control of native NPR1 promoter. NLS-deleted constructs were transiently expressed in mesophyll protoplast of wild-type (WT) tobacco plants. (**b**) CLSM images of GFP fluorescence in mesophyll protoplasts from plants transiently expressing *pNPR1::NPR1(ΔNLS)-GFP* under salt stress. Images of (green) GFP fluorescence and (magenta) chlorophyll autofluorescence are merged in the fourth column. DAPI staining (blue) images are presented in the third column. N indicates NLS.

Figure S7

```
NtNPR1 ----MDNSRTAFSDSNDISGNSSICCIGGGMTEF---FSPETSPAEITSLKRLSETLESIFDASLPEFD-YFADAKLVVPGA---DKEIPVHRCILSARSPFFKNLFCGK--------KE 101
AtNPR1 ----MDTTIDGFADSYEISSTSFVATDNTDSSIVYLAAEQVLTGPDVSALQLLSNSFESVFDS--P--DDFYSDAKLVLSD----GREVSFHRCVLSARSSFFKSALAAAKKEK--DSNN 106
CaNPR1 -----MDSRTAFSDSNDISGSSSICCIG-GMTES---FSPETSPAEITSLKRLSEILESIFDSSSPDFD-FFADAKLVVPI----GKEIPVHRCILSARSPFFKNVFCGK--------ER  98
VvNPR1 -----MDYRAALSDSNDFSGSSSICCIAATTESL----SSEVSPPDISALRRLSENLESVFES--PEFD-FFTDARIVV-AG---GREVPVHRCILAARSVFFKAVLAGA--------RK  96
TcNPR1 -----MDNRNGFSDSNEISNNSSTCCIAAATNSETLASSEPLNTPDIAALQILSRNLESVFES--TDSDSLYSDAKIGL-SS---GREVPVHRCILSARSSVFKTVFSGL---------K 100
OsNPR1 MEPPTSHVTNAFSDSDSA---------------SVEEGGADADADVEALRRLSDNLAAAFRS--PEDFAFLADARIAVPGGGGGGDLLVHRCVLSARSPFLRGVFARRAAAAAGGGGE 102
AtNPR3 -----MATLTEPSSSLSFTSS-HFSYGSIGSNH---FSSSSASNPEVVSLTKLSSNLEQLLSN--S--DCDYSDAEIIVD-----GVPVGVHRCILAARSKFFQDLFKKEKK----IS-K  97
AtNPR4 ----MAATAIEPSSSISFTSS-HLSNPSPVVTT---YH-------SAANLEELSSNLEQLLTN--P--DCDYTDAEIIIEEE---ANPVSVHRCVLAARSKFFLDLFKKDKD----S--S  92
GmNPR1 -----MAYSAEPSSSLSFTSSSHLSNGSVSHNICPSYGSDPGPNLEAISLSKLSSNLEQLLIE--P--DCDYSDADLVVE-----GIPVSVHRCILASRSKFFHELFKREKG----SSEK 102
MhNPR1 -----MAHSAEPSSSLSFTSSPHLSNGSISHNLSC-SGSESVPSLEVISLSKLSSSLEQLLID--P--GCDYSDADIVVE-----GIPVGVHRCILASRSGFFRELFRREKG----SSGK 101

NtNPR1 KNSSKVELKDV----MKEYEVSYDAVVSVLAYLYSGKVRPSPKDVCVCVDNECSHVACRPAVAFLVEVLYTSFTFQISELVDKFQRHLLDILDKAAADDVMMVLSVANICGKACDRLLSS 217
AtNPR1 TAAVKLELKEI----AKDYEVGFDSVVTVLAYVYSSRVRPPPKGVSECADENCCHVACRPAVDFMLEVLYLAFIFKIPELITLYQRHLLDVVDKVVIEDTLVILKLANICGKACMKLLDR 222
CaNPR1 K--TKLELKEL----MKEYEASYDALVNVLAYLYSGKVRPSPKDVCVCVDNECFHVACRPAVAFLVQVLYASFTFQISELVDKFQRHLLDILNKAAADDVMMVLSVANICGKACERLLSS 212
VvNPR1 EKEAKFELKDL----AKEFDVGYDSLVAVLGYLYSGRVGALPKGVCACVDDDCPHSACRPAVDFMVEVLYASFAFQISELVGLYQRRLMDILDKVASDDILVILSVANLCGKACDRLLAR 212
TcNPR1 DRGAKFELKEL----ARDYEIGYDSLVAVLAYLYSGRVRSLPRGVCVCVDDDCSHLACRPAVDFVAEVLYAAFTFQVSELISLYQRHLLDIIDKVEMDDILVVLYVANMCGNTCERLLAK 216
OsNPR1 DGGERLELRELLGGGGEVEVGYEALRLVLDYLYSGRVGDLPKAACLCVDEDCAHVGCHPAVAFMAQVLFAASTFQVAELTNLFQRRLLDVLDKVEVDNLLLILSVANLCNKSCMKLLER 222
AtNPR3 TEKPKYQLREMLPY----GAVAHEAFLYFLSYIYTGRLKPFPLEVSTCVDPVCSHDCCRPAIDFVVQLMYASSVLQVPELVSSFQRRLCNFVEKTLVENVLPILMVAFNCKL--TQLLDQ 211
AtNPR4 EKKPKYQMKDLLPY----GNVGREAFLHFLSYIYTGRLKPFPIEVSTCVDSVCAHDSCKPAIDFAVELMYASFVFQIPDLVSSFQRKLRNYVEKSLVENVLPILLVAFHCDL--TQLLDQ 206
GmNPR1 EGKLKYNMNDLLPY----GKVGYEAFLIFLGYVYTGKLKPSPMEVSTCVDNVCAHDACRPAINFAVELMYASSIFQIPELVSLFQRRLLNFIGKALVEDVIPILTVAFHCQS--NQLVNQ 216
MhNPR1 EDRPKYCMSDFLPY----GDVGYEAFLVFLSYVYTGKLKPSPVEVSTCVHNVCAHDACRPAINFVVELMYAASIFQMPDLVSIFERRLLNFVGKALSDNVVPILLVAFHCQL--NQLIDQ 215

NtNPR1 CIEIIVKSNVDIITLDKALPHDIVKQITDSRAELGLQGPES----NGFPDKHVKRIHRALDSDDVELLQMLLREGHTTLDDAYALHYAVAYCDAKTTAELLDLALADVNHQNSRGYTVLH 333
AtNPR1 CKEIIVKSNVDMVSLEKSLPEELVKEIIDRRKELGLEVPKV--------KKHVSNVHKALDSDDIELVKLLLKEDHTNLDDACALHFAVAYCNVKTATDLLKLDLADVNHRNPRGYTVLH 334
CaNPR1 CIEIIVKSNVDIITLDKALPNDIVKQITDSRTELDLQGPVN----HGFPDKHVKRIHRALDSDDVELLRMLLKEGHTTLDDAYALHYAVAYCDAKTTSELLDLALADVNHQNPRGYTVLH 328
VvNPR1 CIDIIIKSDVDVVTLERALPQEMVKQIVDSRLELGFEEPES----TNFPDKHVKRIHRALDSDDVELVRMLLKEGHTTLDDAYALHYAVAFGDAKTTTELLDLGLADVNHKNHRGYTVLH 328
TcNPR1 CIETLVKSDVDIVTLDKALPYHIVKQIMDSRLELGLDKPEN----TGFPDKHVKRIHRALDSDDVELARMLLKEGHTNLDEASALHYAVAYCDAKTTTELLDLGLADVNRRNSRGYTVLH 332
OsNPR1 CLDMVVRSNLDMITLEKSLPPDVIKQIIDARLSLGLISPEN----KGFPNKHVRRIHRALDSDDVELVRMLLTEGQTNLDDAFALHYAVEHCDSKITTELLDLALADVNHRNPRGYTVLH 338
AtNPR3 CIERVARSDLYRFCIEKEVPPEVAEKIKQLRLISPQDEETSPKI-SEKLLERIGKILKALDSDDVELVKLLLTESDITLDQANGLHYSVVYSDPKVVAEILALDMGDVNYRNSRGYTVLH 330
AtNPR4 CIERVARSDLDRFCIEKELPLEVLEKIKQLRVKSV----NIPEV-EDKSIERTGKVLKALDSDDVELVKLLLTESDITLDQANGLHYAVAYSDPKVVTQVLDLDMADVNFRNSRGYTVLH 321
GmNPR1 CIDRVARSDLDQISIDQELPHELSQKVKLLRRKPQQDVENDASVVDALSLKRITRIHKALDSDDVELVKLLLNESDITLDEANALHYAAAYCDPKVVSEVLGLGLANVNLRNSRGYTVLH 336
MhNPR1 CVDRVARSDINDISLEKGLPDEVVKKIKILRRNYQQDSDPNLSPADPLHEKRIRRIHKALDSDDVELVKPLLTESNITLDEANALHYAAAYCDPKVVTEVLALGLADVNLRNSRGYTVLH 335

NtNPR1 VAAMRKEPKTIVSLLTKGARPSDLTSDGRKALQIAKRLTRLVDFSKSPEEGKSASKDRLCIEILEQAERRDPLL-GEASVSLAMAGDDLRMKLLYLENRVGLAKLLFPMEAKVAMDIAQV 452
AtNPR1 VAAMRKEPQLILSLLEKGASASEATLEGRTALMIAKQATMAVECNNIPEQCKHSLKGRLCVEILEQEDKREQIP-RDVPPSFAVAADELKMTLLDLENRVALAQRLFPTEAQAAMEIAEM 453
CaNPR1 VAAMRKEPKIIVSLLTKGARPSDLTSDGRKALQIAKRRTRLVDFIKSTEEGKSAPKDRLCIEILEQAERRDPLL-GEASVSLAMAGDDLRMKLLYLENRVGLAKLLFPMEAKVAMDIAQV 447
VvNPR1 IAAMRKEPKIIVSLLTKGARPTDITPDGRNALQIAKRLTRAVDYHKSTEEGKPSPKDQLCVEVLEQAERRDPLI-GEASFSLAIAGDDLRMKLLYLENRVGLAKLLFPMEAKVAMDIAQV 447
TcNPR1 VAAMRKEPKIIVSLLTKGARPSDLTLDGRKAFQISKRLTRAADYYMSTEEGKASPKDRLCVEILEQAERRDPLL-GEASLSLAMAGDDLRMKLLYLENRVGLAKLLFPMEAKVAMDIAKV 451
OsNPR1 IAARRREPKIIVSLLTKGARPADVTFDGRKAVQISKRLTKQGDYFGVTEEGKPSPKDRLCIEILEQAERRDPQL-GEASVSLAMAGESLRGRLLYLENRVALARIMFPMEARVAMDIAQV 457
AtNPR3 FAAMRREPSIIISLIDKGANASEFTSDGRSAVNILRRLTNPKDYHTKTAKGRESSKARLCIDILEREIRKNPMVL-DTPMCSISMPEDLQMRLLYLEKRVGLAQLFFPTEAKVAMDIGNV 449
AtNPR4 IAAMRREPTIIIPLIQKGANASDFTFDGRSAVNICRRLTRPKDYHTKTSR-KEPSKYRLCIDILEREIRRNPLVSGDTPTCSHSMPEDLQMRLLYLEKRVGLAQLFFPAEANVAMDVANV 440
GmNPR1 IAAMRKEPSIIVSLLTKGACASDLTFDGQSAVSICRRLTRPKDYHAKTEQGKETNKDRICIDVLEREMRRNPMA-GDACMSSHTMADDLHMKLLYLENRVAFARLFFPSEAKLAMDIAHA 455
MhNPR1 IAVMRKEPSIIVLLLTKGARASELTSDGQSAVSICRRLTRPKDYHSKAEQGQEANKDRICIDVLEREMRRNPMA-GDASISSQIMPDDLHMELLNLENRVALARLFFPAEAKLAMVIAHA 454

NtNPR1 DGTSEFPLASI-GK-KMANAQRTTVDLNEAPFKIKEEHLNRLRALSRTVELGKRFFPRCSEVLNKIMD---ADDLSEIAYMGNDTAEERQLKKQRYMELQEILSKAFTEDKEEFD-KTNH 566
AtNPR1 KGTCEFIVTSL-EPDRLTGTKRTSPGVKIAPFRILEEHQSRLKALSKTVELGKRFFPRCSAVLDQIMN---CEDLTQLACGEDDTAEKRLQKKQRYMEIQETLKKAFSEDNLELG-NSSL 568
CaNPR1 DGTSEFPLASI-RK-KMADAQRTTVDLNEAPFKMKEEHLNRLMALSRTVELGKRFFPRCSEVLNKIMD---ADDLSEIAYMGNDTPEERQLKKQRYMELQEILTKAFTEDKEEFA-KTN- 560
VvNPR1 DGTSEFTLTAI-RPRNLADAQRTTVDLNEAPFRIKEEHLNRLRALSKTVDLGKRFFPRCSEVLNKIMD---ADDLSDLAYLGNGTTEERLLKKRRYKELQDQLCKAFNEDKEEND-KSRI 562
TcNPR1 DGTSEFTLASI-NSNKLNDAQRTTVDLNEAPFRIQEEHLNRLKALSRTVELGKRFFPRCSEVLNKIMD---ADDLSQLACGGNDTPEERLVKKQRYVELQDVLSKAFNEDKVEFD-RSTI 566
OsNPR1 DGTLEFNLGSG--ANPPPERQRTTVDLNESPFIMKEEHLARMTALSKTVELGKRFFPRCSNVLDKIMD---DET--DPVSLGRDTSA---EKRKRFHDLQDVLQKAFHEDKEEND-RSGL 566
AtNPR3 EGTSEFTGLS--PPSSGLTGNLSQVDLNETPHMQTQRLLTRMVALMKTVETGRRFFPYGSEVLDKYMAEYIDDDILDDFHFEKGSTHERRLKRMRYRELKDDVQKAYSKDKESKIARSCL 567
AtNPR4 EGTSECTGLLTPPPSNDTTENLGKVDLNETPYVQTKRMLTRMKALMKTVETGRRYFPSCYEVLDKYMDQYMDEEIPDMSYPEKGTVKERRQKRMRYNELKNDVKKAYSKDK---VARSCL 557
GmNPR1 ETTSEFAGLS-ASNSKGSNGNLREVDLNETPIVQNKRLLSRMEALTKTVEMGRRYFPHCSEVLDKF----MEDDLPDLFYLEKGTHEEQRIKRTRFMELKDDVHKAFNKDKAEFS-RSGI 569
MhNPR1 ETSE-FA--A-PSSSKGSSGNLMEVDLNETPTVQSKRLHSRLEALMKTVRLGRCYFPHCSEVLDKF----IDDDLPHLFYLEPGSSDEQKVKRRRFMELKEEVQKAFDKDKAECN-LSGL 565

NtNPR1 LSS-SCSSTSKGVDKPNKLPFRK---- 588
AtNPR1 TDSTSSTSKSTGGKRSNRKLSHRRR-- 593
CaNPR1 VSS-SCSSTSKGVDKPNKLPFRK---- 582
VvNPR1 -SS-SSSSTSLGFGRNNSRLSCKK--- 584
TcNPR1 SS-SSSSKSIGVSRPNGKLTGSGRGG 591
OsNPR1 -SS-SSSSTSIGAIRPRR--------- 582
AtNPR3 SASSSPSSSSIRDDLHNTT-------- 586
AtNPR4 SSSSPA--SSLREALENPT-------- 574
GmNPR1 SSSSS--SSSLRDSVVHYKARKV---- 590
MhNPR1 PSSSS--TTSPKKIGANQKVREP---- 586
```

Figure S7. Alignment of nonexpressor of pathogenesis-related genes 1 ((NPR1) proteins from different plant species: *Nicotiana tabacum* (NtNPR1, KY402167), *Arabidopsis thaliana* (AtNPR1, ATU76707; AtNPR3, NM_123879.3: AtNPR4, AY785951), *Theobroma cacao* (TcNPR1, HM117159), *Oryza sativa* (Japonica cultivar-group) (OsNPR1, DQ450948), *Glycine max* (GmNPR1, NM001251729), *Capsicum annuum* (CaNPR1, DQ648785), *Vitis vinifera* (VvNPR1, XM002281439), and *Malus hupehensis* (MhNPR1, FJ598141). Two conserved protein–protein interaction motifs, a broad complex, tramtrack, bric a brac/poxvirus and zinc-finger (BTB/POZ)-like domain (pink bar), and an ankyrin-repeat domain (purple bar) are indicated. Nuclear localization signal is indicated with asterisks and a green open box. Two cysteines (Cys521 and Cys529) required for salicylic acid (SA)-dependent transcriptional activation in *Arabidopsis* are indicated in uppercase C in red. The putative NIMIN1 and NIMIN2 binding regions are indicated with asterisks and a black open box. The SA-binding region is indicated with asterisks and a red open box. Specific amino acids playing important roles in SA binding are indicated with red arrows. The conserved region (VDLNE) of ethylene-responsive element binding factor-associated amphipathic repression motif is indicated with a blue bracket. Red box (■) in *Arabidopsis* NPR1 C156 indicates the redox sensor for oligomerization.

Figure S8

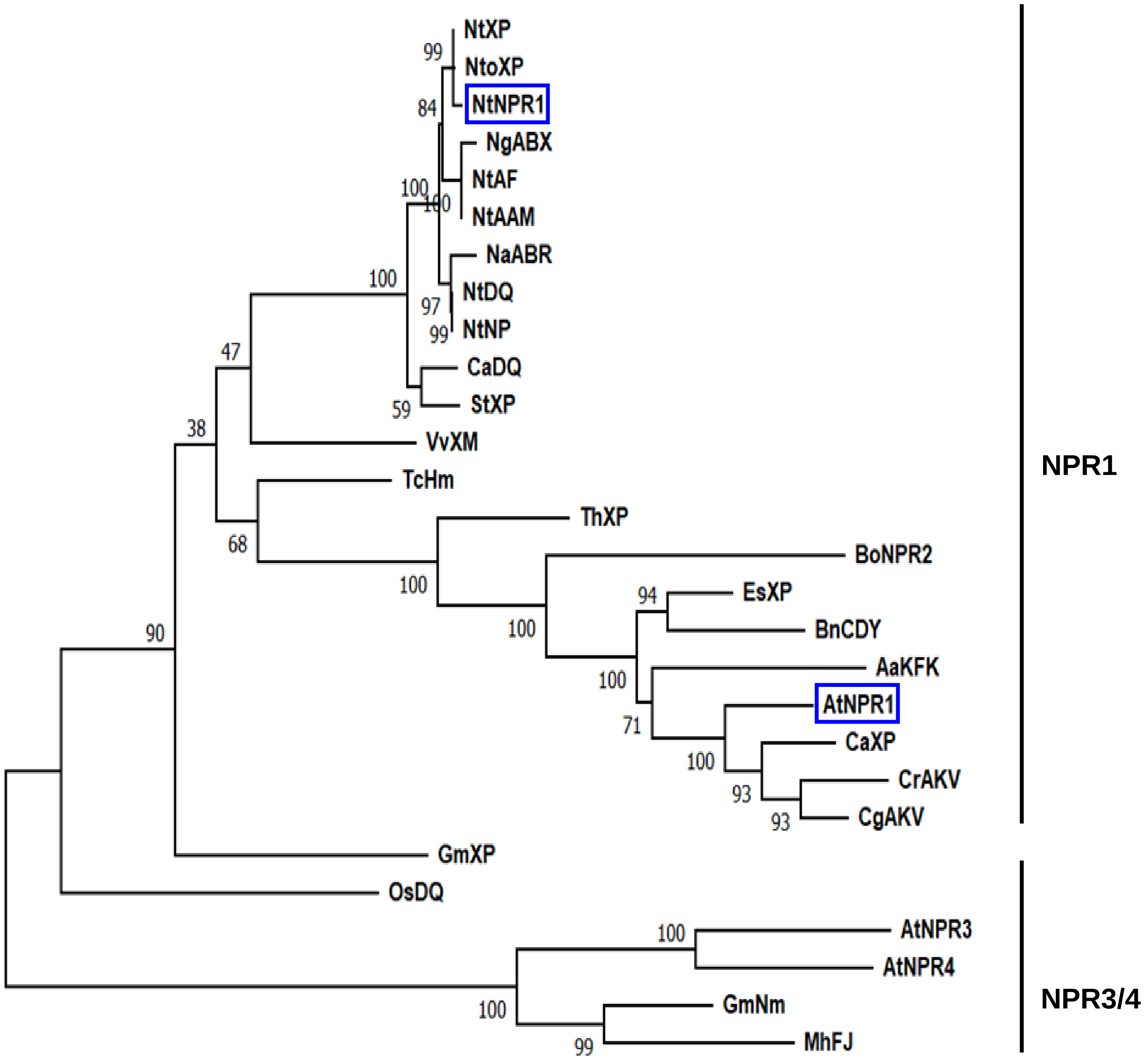
NtXP
NtoXP
NtNPR1
NgABX
NtAF
NtAAM
NaABR
NtDQ
NtNP
CaDQ
StXP
VvXM
TcHm
ThXP
BoNPR2
EsXP
BnCDY
AaKFK
AtNPR1
CaXP
CrAKV
CgAKV
GmXP
OsDQ
AtNPR3
AtNPR4
GmNm
MhFJ
99
84
100
97
99
59
47
38
68
100
100
94
100
71
100
93
93
90
100
100
99
NPR1
NPR3/4

Figure S8. Phylogenetic tree constructed using the neighbor-joining method in MEGA version 11.0 based on the sequences of 28 NPR1 genes from different plants species. Amino acid sequence of each protein was downloaded from NCBI. Each number at nodes is the percentage of 1,000 bootstrap replicate support. Abbreviations and GenBank accession numbers are presented in parentheses after the species name: *Nicotiana tabacum* (NtNPR1, KY402167; NtAF, AF480488; NtAAM, AAM62410.1; NtXP, XP_016503804; NtNP, DQ837218.1 and NtDQ, DQ837218), *Nicotiana glutinosa* (NgABX, ABX71071.1), *Nicotiana tomentosiformis* (NtoXP, XP_009606361), *Nicotiana attenuate* (NaABR, ABR23001.1), *Solanum tuberosum* (StXP, XM_006357647), *Arabidopsis thaliana* (AtNPR1, ATU76707; AtNPR3, NM_123879.3: AtNPR4, AY785951), *Theobroma cacao* (TcHM, HM117159), *Oryza sativa* (japonica cultivar-group) (OsDQ, DQ450948), *Glycine max* (GmNM, NM001251729; GmXP, XP_003534926), *Capsicum annuum* (CaDQ, DQ648785), *Vitis vinifera* (VvXM, XM002281439), *Camelina sativa* (CaXP, XP_010430551), *Capsella rubella* (CrAKV, AKV91676.1), *Capsella grandiflora* (CgAKV, AKV91675.1), *Eutrema salsugineum* (EsXP, XP_006391648.1), *Brassica napus* (BnCDY, CDY34413.1), *Arabis alpine* (AaKFK, KFK36581.1), *Tarenaya hassleriana* (ThXP, XP_010545358.1), *Brassica oleracea* var. *oleracea* (BoNPR2, XM_013780436), and *Malus hupehensis* (MhFJ, FJ598141).

Figure S9

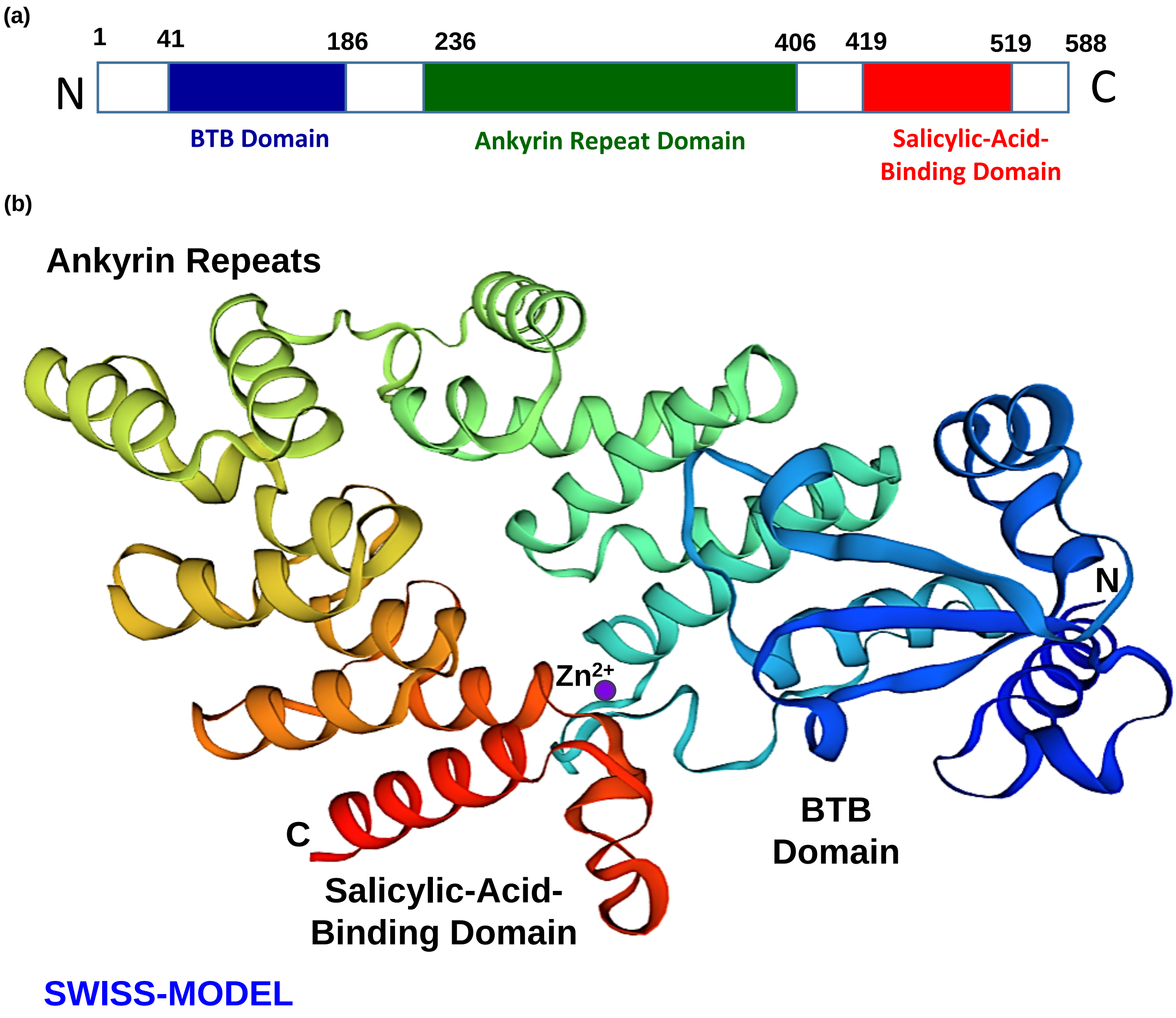

Figure S9. Information of functional domains and homology model NPR1 protein from *Nicotiana tabacum* (NtNPR1, KY402167) for functional analysis. (**a**) Domain map of the NtNPR1 protein to BTB domain, ankyrin repeats domain, and salicylic acid-binding domain using InterProscan for identifying the functional domains and protein families. (**b**) An NtNPR1 homology model (43–405 aa) was created with SWISS-MODEL at the highest score of global model quality estimate with the CryoEM structure of *Arabidopsis* NPR1.

Figure S10

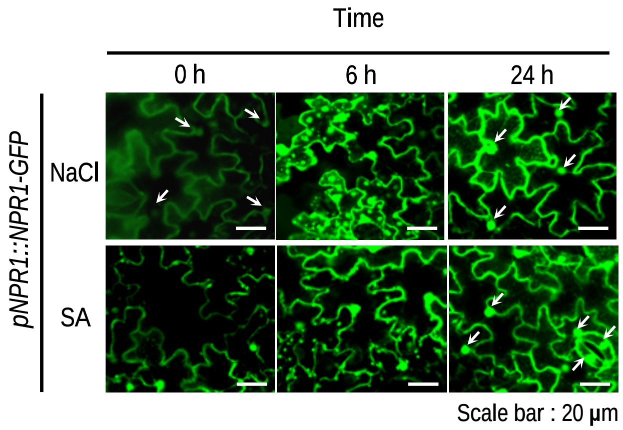

Figure S10. GFP fluorescence in the chloroplasts and nucleus of *p35S::cTP-NPR1-GFP* transgenic tobacco plants under salt stress. GFP-tagged nonexpressor of pathogenesis-related gene 1 (NPR1–GFP) localization in the pavement cells of abaxial leaf epidermis in *pNPR1::NPR1–GFP* transgenic plants under salt stress (upper) and salicylic acid (SA) treatment (middle) and *p35S::cTP-NPR1-GFP* transgenic plants under salt stress (lower). Arrows indicate NPR1 condensates.

Figure S11

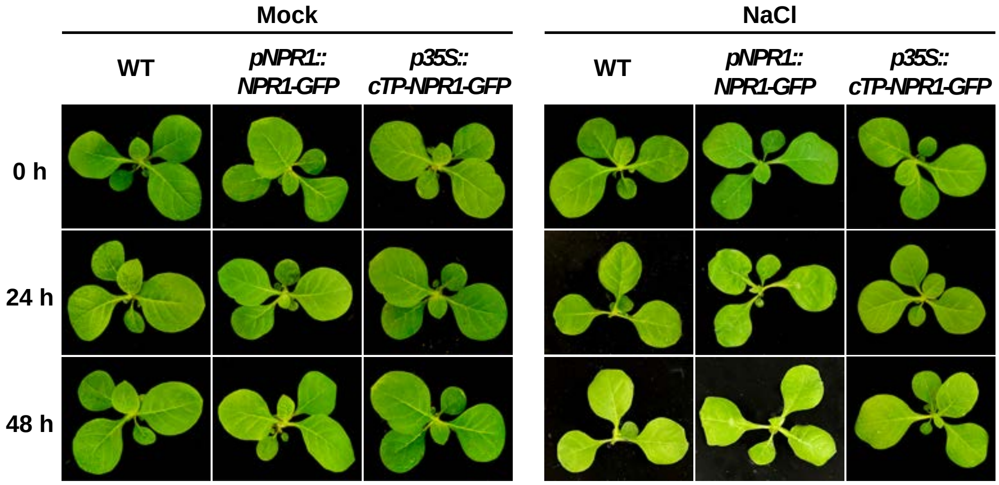

Figure S11. Phenotypes of WT and two transgenic tobacco lines under salt stress. Whole tobacco plants (WT, *pNPR1::NPR1-GFP*, and *p35S::cTP-NPR1-GFP*) were exposed to salt stress for 48 h.

Figure S12

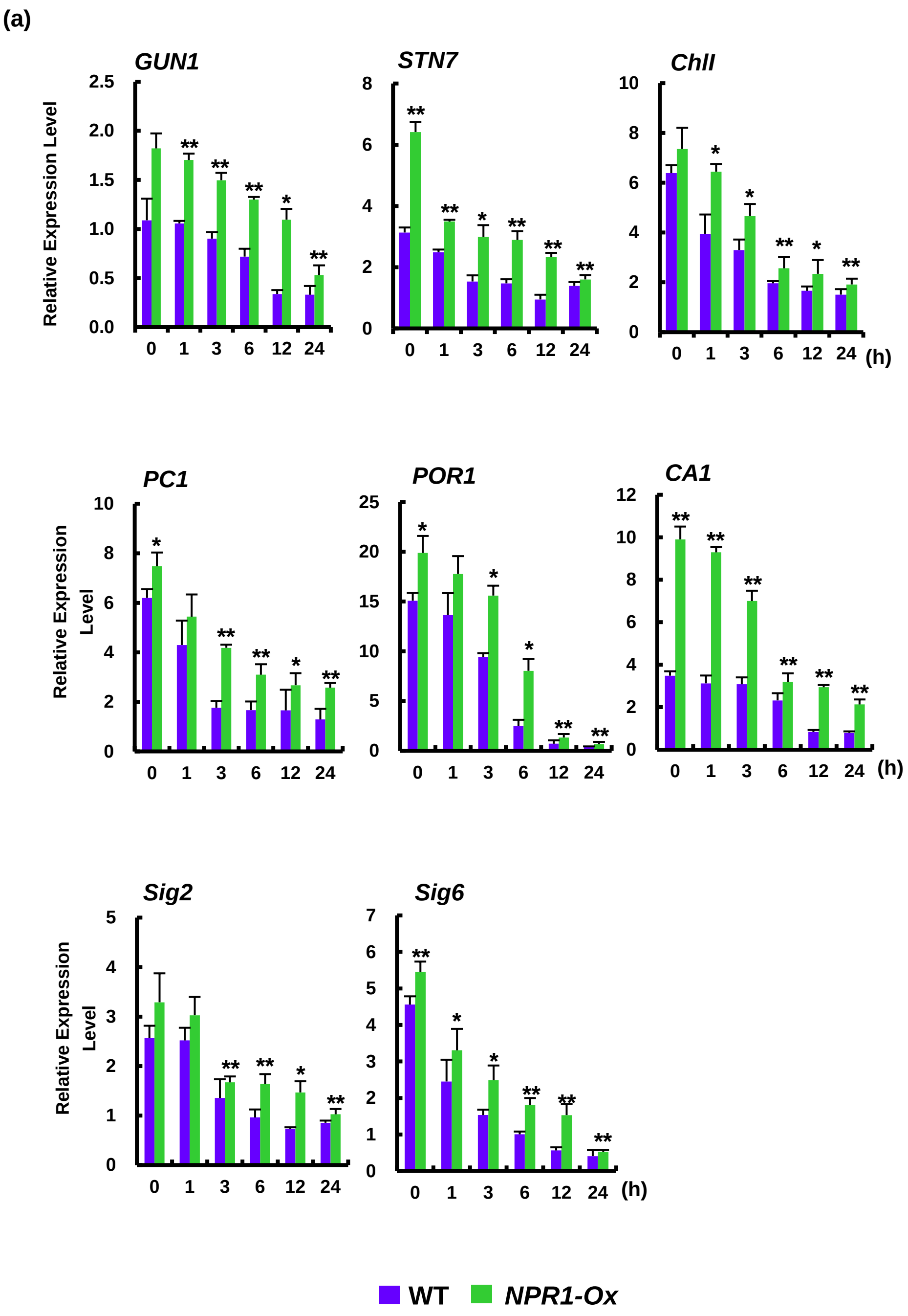
(a)
GUN1
STN7
ChlI
PC1
POR1
CA1
Sig2
Sig6
Relative Expression Level
0 1 3 6 12 24 (h)
WT
NPR1-Ox

(b)

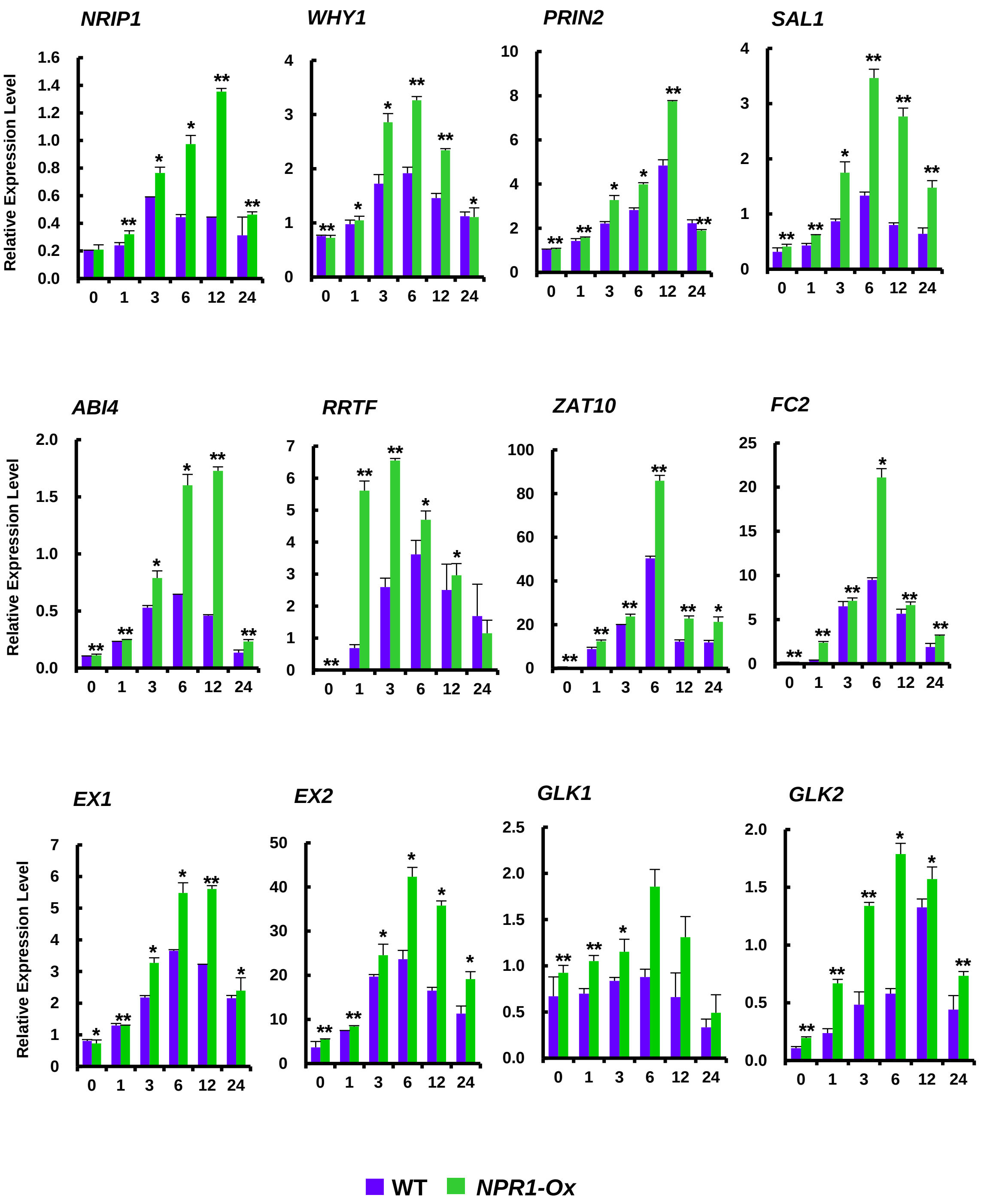
NRIP1
WHY1
PRIN2
SAL1
ABI4
RRTF
ZAT10
FC2
EX1
EX2
GLK1
GLK2
Relative Expression Level
0 1 3 6 12 24
WT
NPR1-Ox

Figure S12. Transcription of retrograde signaling-related genes involved in chloroplast development and abiotic/biotic stress response in WT and NPR1 overexpression (*NPR1-Ox*) transgenic tobacco plants under salt stress. Transcription profiles were determined using qRT-PCR in response to salt stress with 200 mM NaCl in WT and *NPR1-Ox* transgenic plant leaves and expressed relative to that of the reference gene β-actin. (**a**) Genes related to retrograde communication for chloroplast development: *GUN1*, genomes uncoupled1; *STN7*, serine/threonine-protein kinase7; *ChlI*, magnesium–protoporyphyrin chelatase subunit; *PC1*, plastocyanin; NADPH:*POR1*, protochlorophyllide oxidoreductase; *CA1*, carbonic anhydrase 1; *Sig2*, chloroplast sigma factor 2; *Sig6*, chloroplast sigma factor 6. (**b**) Genes related to retrograde communication for operational signaling in response to biotic/abiotic stress: *NRIP1*, chloroplast NB-LRR receptor-interacting protein 1; *WHY1*, single-stranded DNA-binding protein WHIRLY 1; *PRIN2*, plastid redox insensitive 2; *SAL1*, 3′-phosphoadenosine 5′-phosphate (PAP) phosphatase; *ABI4*, abscisic acid-insensitive protein 4; *RRTF*, redox-responsive transcription factor; *ZAT10*, zinc-finger transcription factor 10; *FC2*, ferrochelatase; *EX1*, executer1; *EX2*, executer2; *GLK1*, golden 2-like1; *GLK2*, golden 2-like 2. Relative transcript expression levels are presented as mean ± SD. Asterisks indicate significant differences between the WT and transgenic plants in each case (**P*<0.05 or ***P*<0.01).

Figure S13

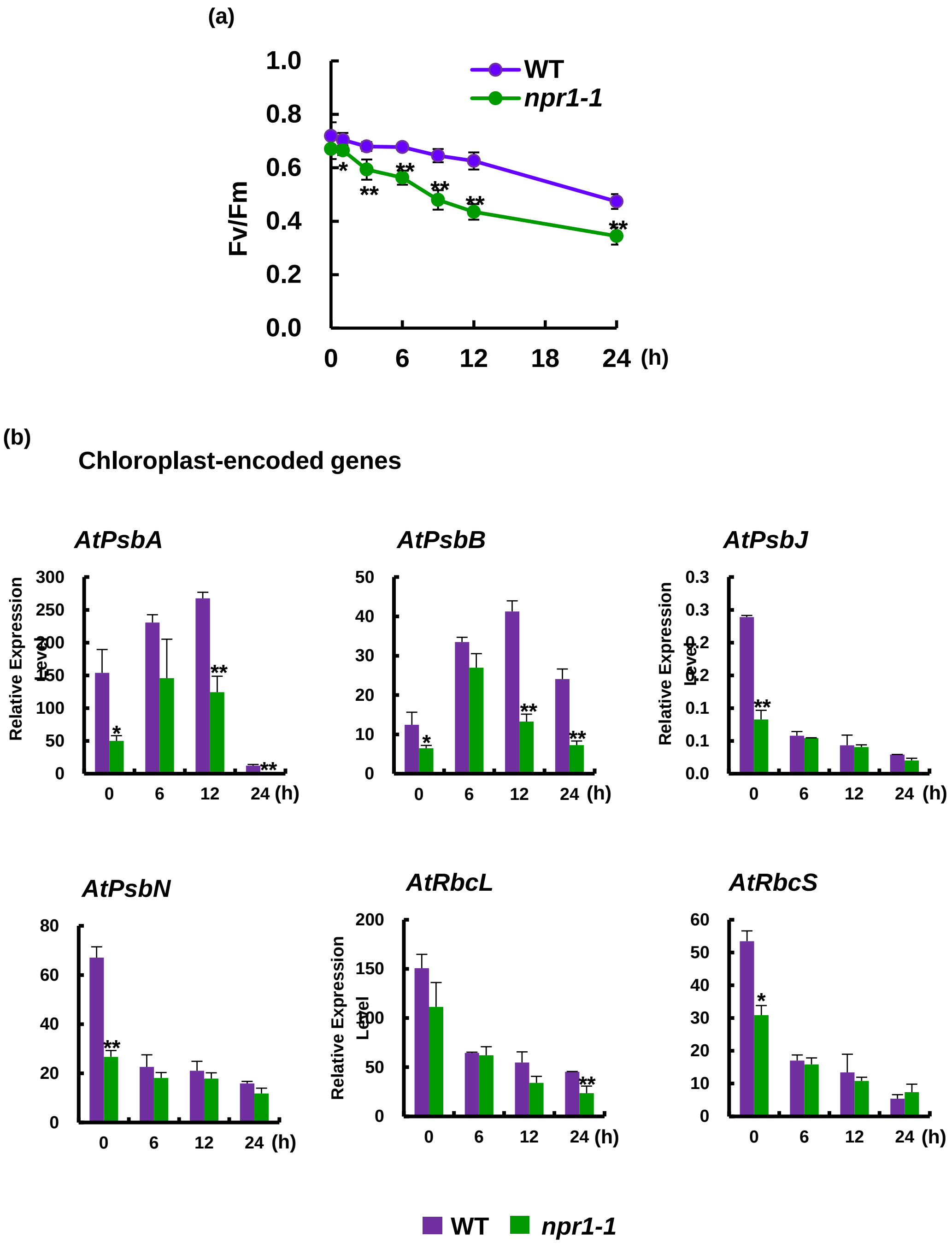
(a)
WT
npr1-1
Fv/Fm
1.0
0.8
0.6
0.4
0.2
0.0
0
6
12
18
24 (h)
*
**
(b)
Chloroplast-encoded genes
AtPsbA
AtPsbB
AtPsbJ
AtPsbN
AtRbcL
AtRbcS
Relative Expression Level
0
6
12
24 (h)
WT
npr1-1

Figure S13. Kinetics of maximal photochemical efficiency and chloroplast-encoded gene transcription in WT and *npr1-1* mutant *Arabidopsis* plants under salt stress. (**a**) Maximal photochemical efficiency of photosystem II (PC II) (*Fv*/*Fm*) was measured in WT and *npr1-1* mutant *Arabidopsis* plants under salt stress. *Fv*/*Fm* values are expressed as mean ± SD. (**b**) Transcription levels in WT and *npr1-1* mutant *Arabidopsis* plants in response to 200 mM NaCl-induced salt stress were measured using qRT-PCR and expressed relative to that of the reference gene β-actin. Chloroplast-encoded genes: *AtPsbA*, photosystem II reaction center D1 protein; *AtPsaB*, *AtPsbJ*, PC II reaction center protein J; *AtPsbN*, PC II subunit; *AtRbc L*, RuBisCo large subunit; *AtRbc S*, RuBisCo small subunit. Relative transcript expression levels are presented as mean ± SD. Asterisks indicate significant differences between WT and *npr1-1* mutant *Arabidopsis* plants at the indicated time points (*P<0.05 or **P<0.01).

Figure S14

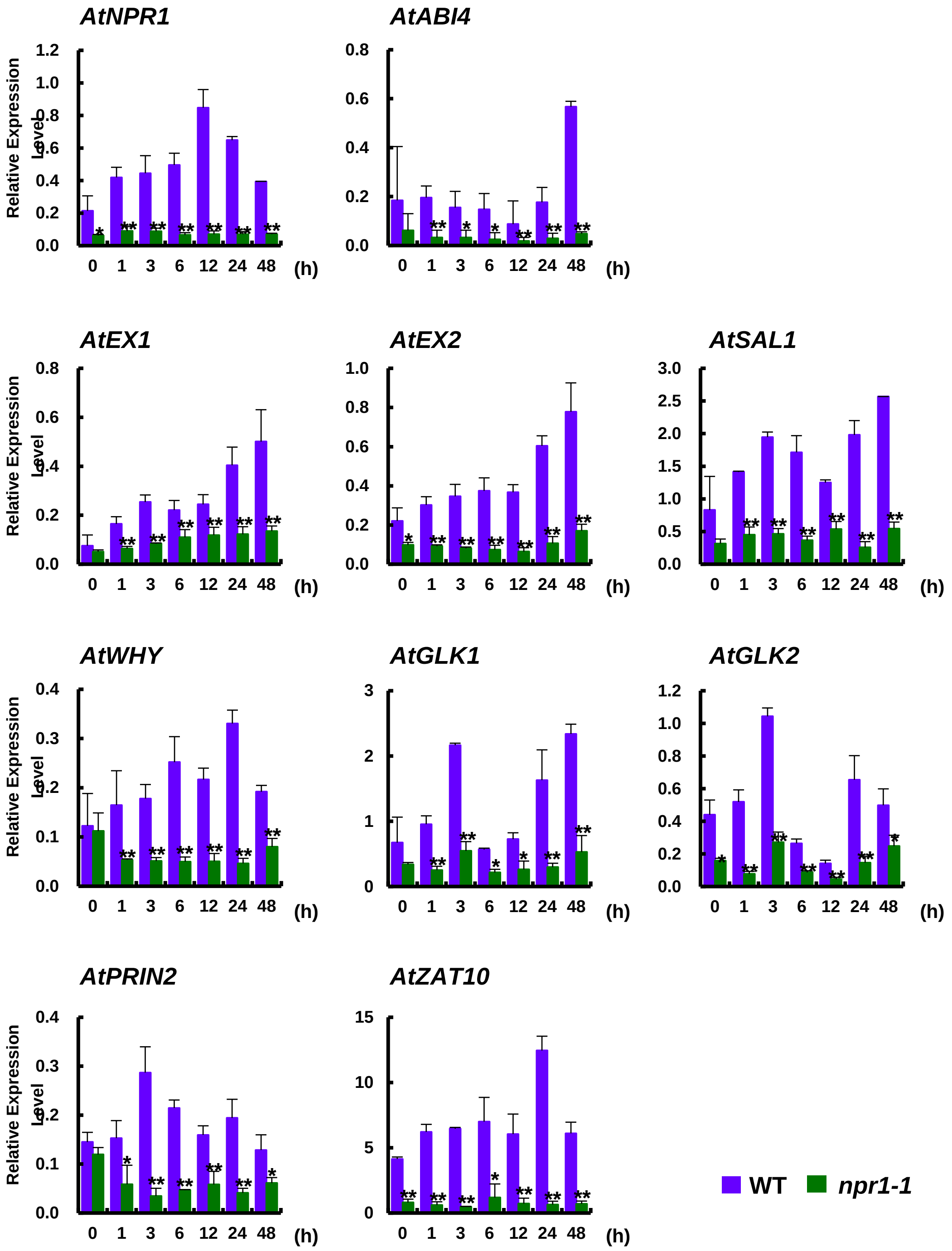

AtNPR1
AtABI4
AtEX1
AtEX2
AtSAL1
AtWHY
AtGLK1
AtGLK2
AtPRIN2
AtZAT10
Relative Expression Level
0 1 3 6 12 24 48 (h)
WT
npr1-1

Figure S14. Transcription of retrograde signaling-related genes in response to abiotic/biotic stress in Col-0 WT and *npr1-1* mutant *Arabidopsis* plants under salt stress. Transcription profiles in the leaves of WT and *npr1-1* mutants in response to 200 mM NaCl-induced salt stress were determined using qRT-PCR and expressed relative to that of the reference gene β-actin. Genes related in retrograde communication for operational signaling in response to biotic/abiotic stress: *AtNPR1*, nonexpressor of pathogenesis-related genes 1; *AtABI4*, abscisic acid-insensitive protein 4; *AtEX1*, executer1; *AtEX2*, executer2; *AtSAL1*, 3′-phosphoadenosine 5′-phosphate (PAP) phosphatase; *AtWHY1*, single-stranded DNA-binding protein WHIRLY 1; *AtGLK1*, golden 2-like1; *AtGLK2*, golden 2-like 2; *AtPRIN2*, plastid redox insensitive 2; *AtZAT10*, zinc-finger transcription factor 10. Relative transcript expression levels are presented as mean ± SD. Asterisks indicate significant differences between WT and *npr1-1* mutant *Arabidopsis* plants at the indicated time points (*$P<0.05$ or **$P<0.01$).

Supplementary materials

The full scan of the entire original blots

The full scan of the entire original blots: Figure 1A

Chloroplast

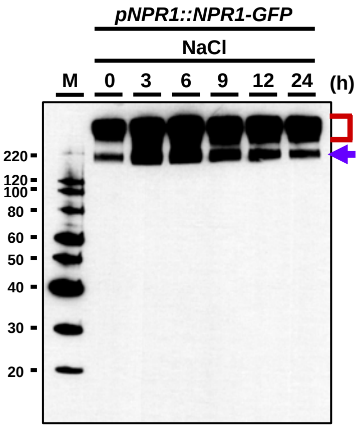

Ab: α-GFP
protein: 10μg

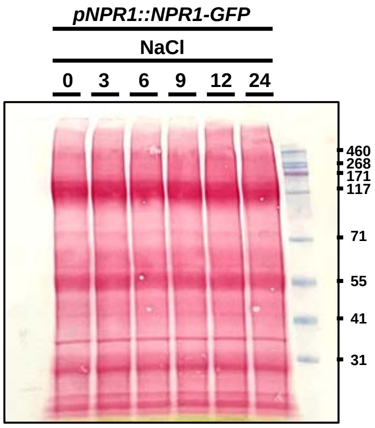

Ponceau S
protein: 10μg

Relative Intensity

oligomer	1.0	1.6	1.5	1.4	1.4	1.1
dimer	1.0	2.8	2.6	2.1	1.8	1.1

Nuclear marker: anti-Histon H3 antibody

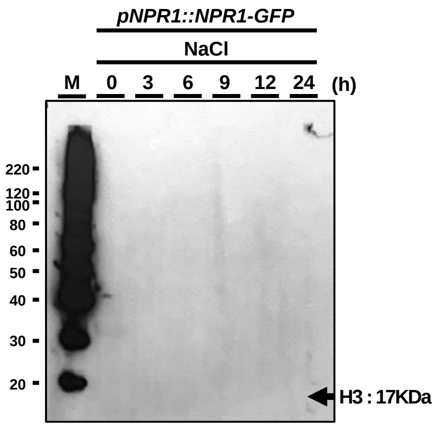

Ab: α-H3
protein: 10μg

Chloroplast stroma protein marker: anti-RbcL antibody

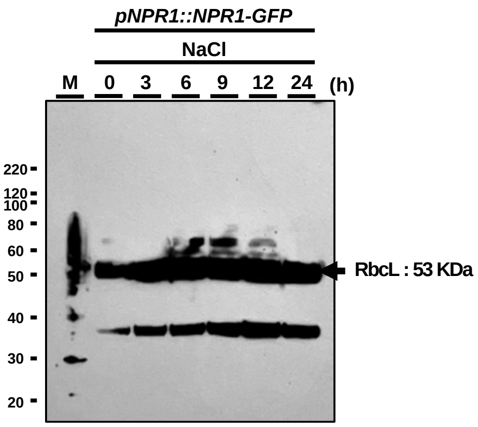

Ab: α-RbcL
protein: 10μg

Chloroplast outer membrane marker: anti-Toc75 antibody

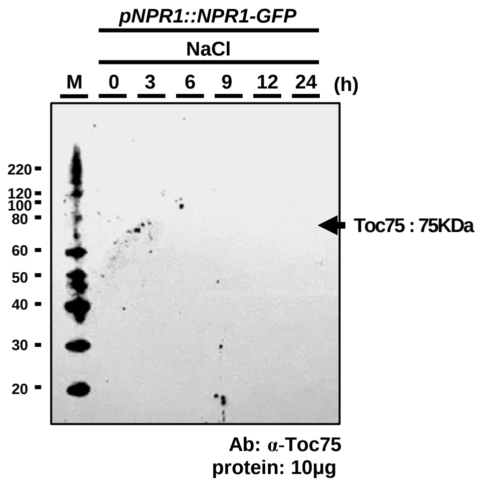

To ensure the reliability of our results, all experiments were repeated at least three times. Data from representative experiments were then reported after confirming consistency in the obtained results. To quantify band intensities, we utilized Image J software. Relative intensity was calculated by normalizing the band intensity values to the 0-hour treatment, which was assigned as 1.0.

The full scan of the entire original blots: Figure 1B

NPR1-GFP

Nucleus

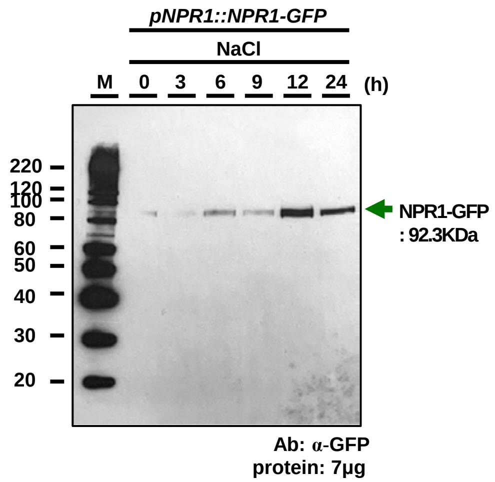

Marker of Nuclear proteins and equal loading: anti-Histon H3 antibody

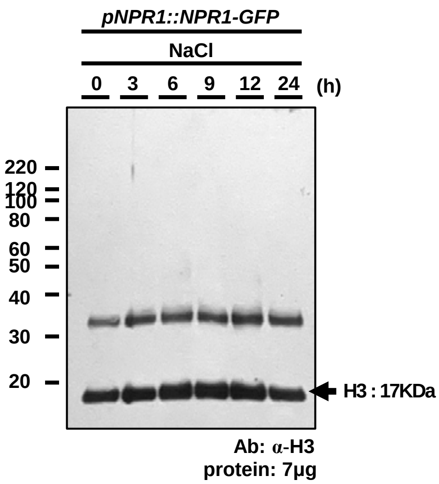

Relative Intensity

monomer 1.0 1.3 7.5 7.0 30.0 12.7

Chloroplast stroma marker: anti-RbcL antibody

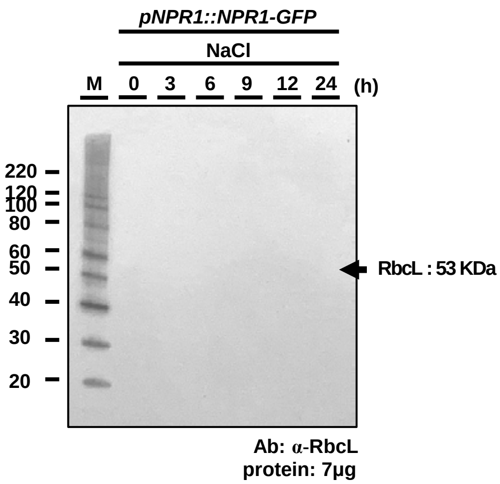

Chloroplast outer membrane marker: anti-Toc75 antibody

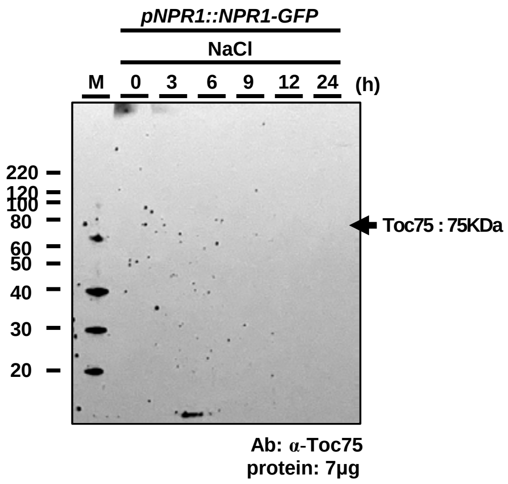

To ensure the reliability of our results, all experiments were repeated at least three times. Data from representative experiments were then reported after confirming consistency in the obtained results. To quantify band intensities, we utilized Image J software. Relative intensity was calculated by normalizing the band intensity values to the 0-hour treatment, which was assigned as 1.0.

The full scan of the entire original blots: Figure 1D

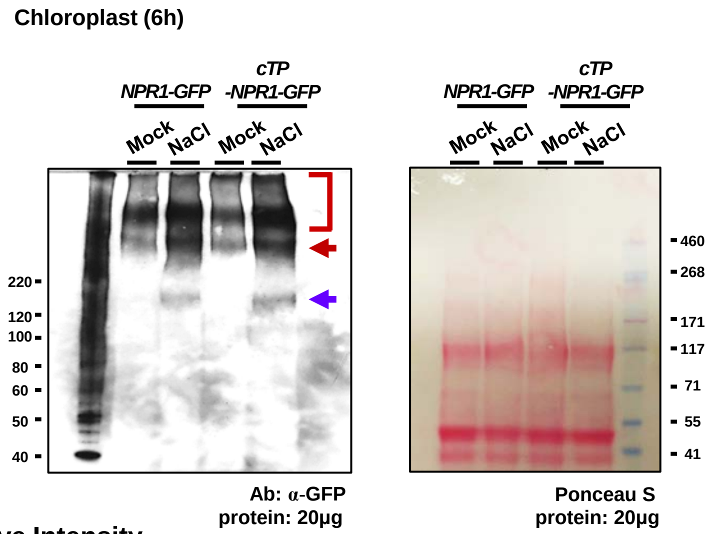

Relative Intensity

oligomer	1.0	1.3	0.7	1.4
tetramer	1.0	2.2	0.9	3.9
dimer	1.0	144.6	1.0	216.2

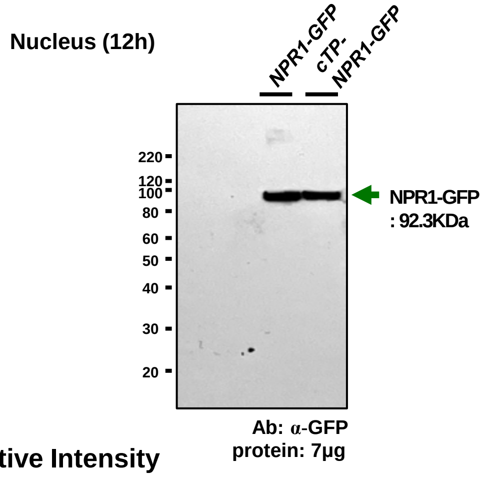

Relative Intensity

monomer	1.0	0.8

To ensure the reliability of our results, all experiments were repeated at least three times. Data from representative experiments were then reported after confirming consistency in the obtained results. To quantify band intensities, we utilized Image J software. (left) Relative intensity was calculated by normalizing the band intensity values to the mock treatment of *pNPR1::NPR1-GFP*, which was assigned a value of 1.0. (right) Relative intensity of *p35S::cTP-NPR1-GFP* was calculated by normalizing the band intensity values to *pNPR1::NPR1-GFP*, which was assigned as 1.0.

The full scan of the entire original blots: Figure 1G and 1J

Chloroplast

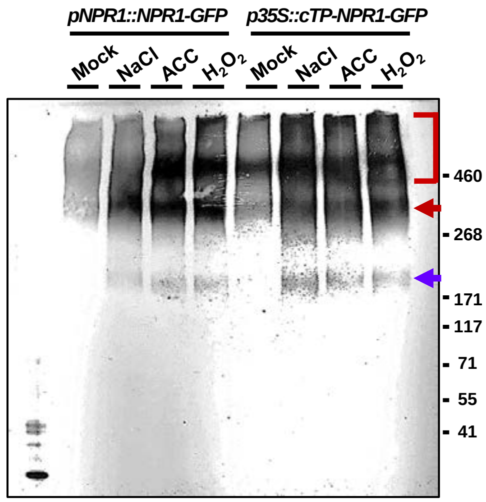

Ab: α-GFP
protein: 20µg

Relative Intensity

oligomer	1.0	1.4	2.0	2.1	2.0	2.5	2.0	2.2
tetramer	1.0	3.3	3.7	3.6	2.2	4.1	4.2	4.1
dimer	1.0	257	264.	209	1.1	538	291	120

Marker of chloroplast stroma proteins : anti-RbcL antibody

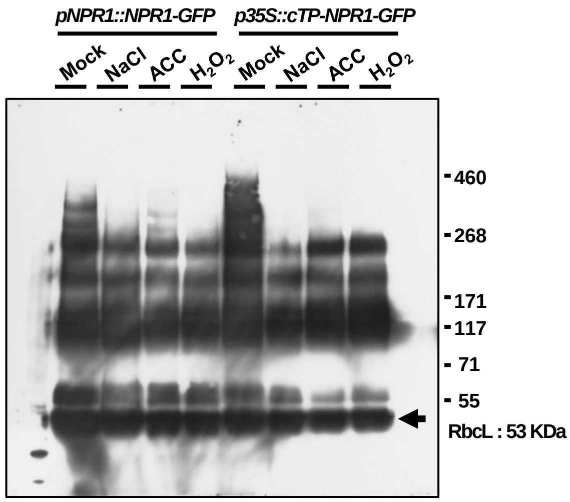

Ab: α-RbcL
protein: 20µg

Nucleus

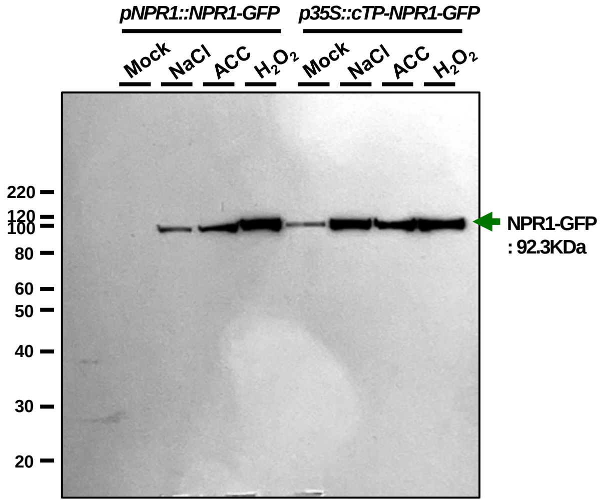

Ab: α-GFP
protein: 7µg

Relative Intensity

monomer	1.0	8.9	22.4	34.6.	1.5	26.6	28.0	40.2

Marker of nuclear proteins and Equal loading: anti-Histon H3 antibody

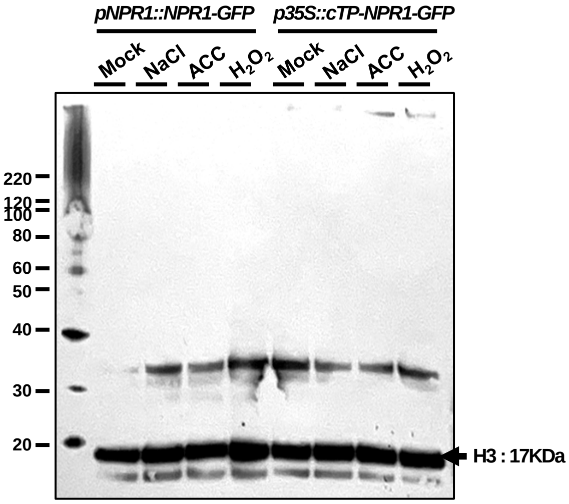

Ab: α-H3
protein: 3µg

To ensure the reliability of our results, all experiments were repeated at least three times. Data from representative experiments were then reported after confirming consistency in the obtained results. To quantify band intensities, we utilized Image J software. Relative intensity was calculated by normalizing the band intensity values to the mock treatment of *pNPR1::NPR1-GFP*, which was assigned as 1.0.

The full scan of the entire original blots: Figure 3D

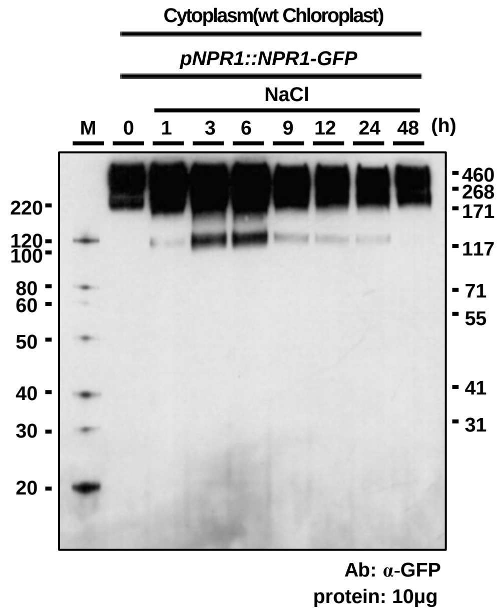

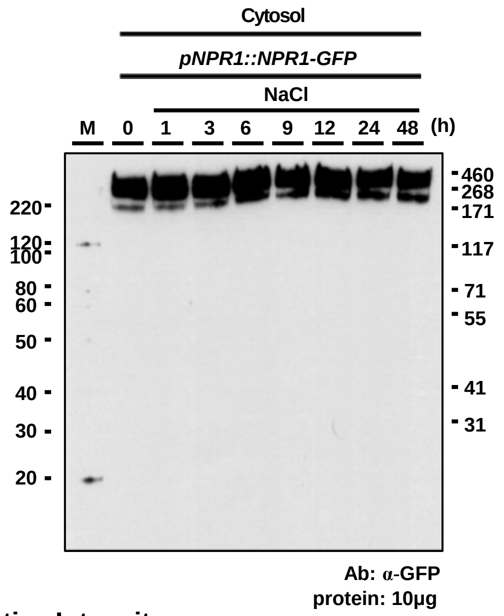

Relative Intensity

oligomer	1.0	1.4	1.6	1.7	1.2	1.1	0.9	0.9
dimer	1.0	21.5	227.9	236.7	33.2	9.6	5.2	0.0

Relative Intensity

oligomer	1.0	1.0	1.0	1.0	0.9	1.0	0.9	0.9
dimer	1.0	1.0	0.9	1.0	1.0	1.1	1.0	1.0

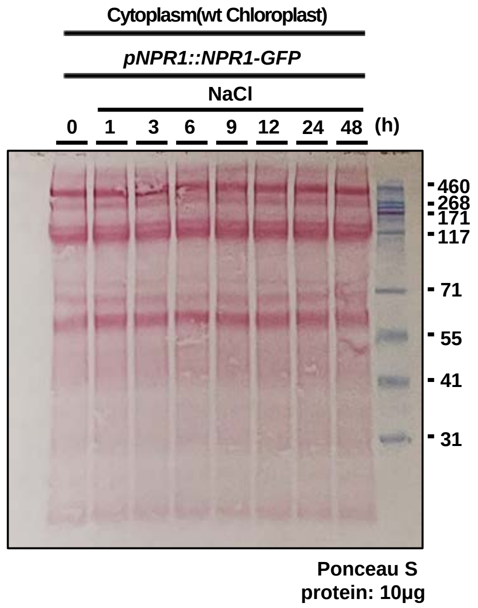

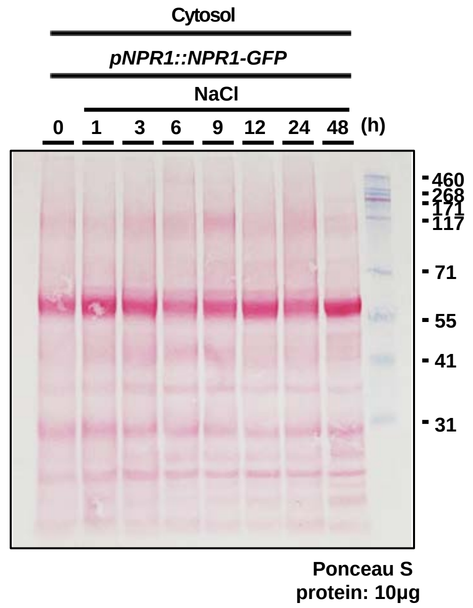

To ensure the reliability of our results, all experiments were repeated at least three times. Data from representative experiments were then reported after confirming consistency in the obtained results. To quantify band intensities, we utilized Image J software. Relative intensity was calculated by normalizing the band intensity values to the 0-hour treatment, which was assigned as 1.0.
