# Supplementary material for: NPR1 Translocation from Chloroplast to Nucleus Activates Plant Tolerance to Salt Stress
Source: Antioxidants (Basel). 2023 May 18;12(5):1118. doi: 10.3390/antiox12051118 (PMC10215103; doi:10.3390/antiox12051118)
Supplement: Supplementary file 1 [file antioxidants-12-01118-s001.zip › Table S1.pdf]

Table S1. Sequences of primers for real-time qRT-PCR

Gene	Strand	Sequences
β-Actin	Forward<br>Reverse	TCACAGAAGCTCCTCCTAATCC<br>GGGAAAGAACAGCCTGAATG
Chl I	Forward<br>Reverse	AGCTCCAGAACCAAATCGAC<br>TCTTGCTGCCCTGTTAGTGA
GUN1	Forward<br>Reverse	TTCGCTGCTTGCTGTTTGTAG<br>AAAAGCAACATCTACCTGCCC
STN7	Forward<br>Reverse	GGGAGCTATTAGCATCTATGG<br>TTGTGTAGCCCGGAAGAATTG
PC1	Forward<br>Reverse	TTCAAGAACAATGCCGGGTTC<br>GCGCACAGTAGAAAGTGTAAG
POR1	Forward<br>Reverse	TGTCCAATGGAGCAGTAAGG<br>CCAGGCCTAATCCTGAAGAG
SIG2	Forward<br>Reverse	AGTTGGAAAGACTTGAGCAG<br>CCATAGTTCAGACGCTTCCTT
SIG6	Forward<br>Reverse	TGTTCTGAGTCCAAAGGAGAG<br>TTTAGCCTGTATAGTGCACGG
NRIP1	Forward<br>Reverse	AAAAATCTCCCAGACCCCAC<br>CTTGGATAACCTGTGGTTGG
WHY1	Forward<br>Reverse	GAGAGGGTTTTGTGCTGCTTC<br>CAACCCTCAACACCTTCCTGAC
PRIN2	Forward<br>Reverse	CTTGCATGCCGAGTATGGTG<br>CATTGTCTACGTGAGTTTGAGCCC
ABI4	Forward<br>Reverse	TTCACAGCAGTTGGTTCAGC<br>TCAAGTTTTGGCGGCTTCTG
ZAT10	Forward<br>Reverse	ACACAAAGCTAGTCACCGGAAG<br>TTACTGTTACCGCCGTCGTAG
EX1	Forward<br>Reverse	TGATGGCGAAGAATGGGGTTG<br>GCAATGCCCACCTTGAGTTTAG
EX2	Forward<br>Reverse	TCAGGTTGGGATTGGAACCG<br>TTCAGCAACAGAGTCCTTCGC
SAL1	Forward<br>Reverse	CTTGCTGCTGCTAAGAAAGCTG<br>GTCCTCCTCAGCCACTAATG
GLK1	Forward<br>Reverse	AGAGATGAATCCGTGGCCTG<br>GTGAAGGATGAAGATGAGGAGG
GLK2	Forward<br>Reverse	TATAGCGGAGCCACCACAATC<br>AGGTGCTGCAAATCTCGCTG
FC2	Forward<br>Reverse	CTCTCGGCTTCTTCAACCAATG<br>GACGATCCACCAAATACACTGC
CA!	Forward<br>Reverse	GCAGGAAGCTGTGAATGTATC<br>GAGAAAGACCGAAGTTGAGTC
RRTF	Forward<br>Reverse	CCTCTGTTTCGCGTTCCAAAAG<br>ATTTTCCCCACGGTCTCTGTC
NPR1	Forward<br>Reverse	GCTGTAGCATATTGCGATGC<br>GCAACATGCAGCACTGTGTA
GFP	Forward<br>Reverse	ACTACCTGTTCCATGGCCAA<br>AAGCTCGATCCTGTTGACGA
PR-1	Forward<br>Reverse	TTGAGATGTGGGTCGATGAG<br>CCTAGCACATCCAACACGAA
PR-2	Forward<br>Reverse	ACTGAGGCACAAGGAGCATA<br>CACGAATAGATGCACCACGG
PR-3	Forward<br>Reverse	AGGAACGACGGTAGATGTCC<br>TCCTACGGGCAGTATCATCA

PR-4	Forward<br>Reverse	ATGGCTGGACTGCTTTCTGT<br>CTCACTGTTGCTTGAGTTCCTG
PR-5	Forward<br>Reverse	GTCGTAATCTCAGATGCACAGC<br>AGTAGGCCCACATGATCCAG
TGA2	Forward<br>Reverse	ACAAGCAGAAGCCCATTTGG<br>TGGCCTTGAACTCCAATTGG
ACS5	Forward<br>Reverse	CATCCACCTCGTATGTGACG<br>CCGACTCTGAATCCAGGAAA
Rbc S	Forward<br>Reverse	AGGTGTGGCCACCAATTAAC<br>GTCTCGAATTCCAAGCAAGG
CAB3	Forward<br>Reverse	ATTGGGTCTTGCTGAAGATCC<br>TTCCGGGAACAAAGTTTGTGG
CAB13	Forward<br>Reverse	GCAATGCTTGGAGCACTAGG<br>CTGAGCATGCACAAGGTTAGG
CAB21	Forward<br>Reverse	GTCCATTCTCCGGTGAGTCC<br>CACATCCAAGAGCACCAAGC
CAB36	Forward<br>Reverse	CATTCGCTAGGAACCGTGAG<br>TATCTGAGATCCGGCCTTGA
PsaF	Forward<br>Reverse	CCATGCAAGGAGTCTAAGCA<br>GCAATCCATCTGATCCACAC
PsaK	Forward<br>Reverse	ATGAGGCGTAAAGGACAAGG<br>GCCTTCCTATTTGCTGATGG
PsaN	Forward<br>Reverse	TTGGTGCATCTGAACTCACC<br>GCTTGGTTCTTGTTGGCTTC
AtNPR1	Forward<br>Reverse	GAATCCGTCTTTGACTCGCC<br>GCGGTGTTGTTGGAGTCTTTC
AtABI4	Forward<br>Reverse	AGATCCGAGAGCCACGTAAG<br>AGGGAGGAGAGGTCTTAGGG
AtEX1	Forward<br>Reverse	GATGATGATTGGGATTGGGG<br>AATTGCCACCTTTAGCCTCG
AtEx2	Forward<br>Reverse	GTTGGAACCGTCACTTCTCC<br>TCAGCGACAGCATCATCAAC
AtSAL1	Forward<br>Reverse	CCGCGAAAAGATTTGGGACC<br>GCTATGGAGTCACGAACTGC
AtWHY	Forward<br>Reverse	GCTGAAGGATTGCCTGCTAG<br>TGTCGTACACCAGCAGAAGG
AtGLK1	Forward<br>Reverse	TTGGATTTGGTGGTGCTCGTC<br>AGCTCTGTCATAACACCGTC
AtGLK2	Forward<br>Reverse	GCCGTCTCGAATCTTGGAAA<br>ATGTCTCCGGAGATTCCAGC
AtPRIN2	Forward<br>Reverse	TCTTGCGCAGTGAGTATGGA<br>GACACATCTGCTACCCCACT
AtZAT10	Forward<br>Reverse	TCACACGTTTGCACCATCTG<br>TTCGTCGTCTCCGTTGACCAT
AtPsbA	Forward<br>Reverse	TATGGGTCGTGAGTGGGAAC<br>GAATGTTGTGCTCAGCCTGG
AtPsbB	Forward<br>Reverse	GATCCCGGTCGTTTACTTGC<br>ATATTCCAACCGCCCCAAGA
AtPsbJ	Forward<br>Reverse	CTGATACTACTGGAAGGATTCC<br>CTACAGGGATGAACCTAATCG
AtPsbN	Forward<br>Reverse	GAAACAGCAACCCTAGTCGC<br>CTAGTCCCCGTGTTCCTCG
AtRbcL	Forward<br>Reverse	AGGGAGTCAACTTTGGGCTT<br>AGGGTGGCCTAAAGTTCCTC
AtRbcS	Forward<br>Reverse	TTCGCAACACTAAGCCTACG<br>TCTCCCATACCTTCTGCGAG
